# Computer anthropomorphisation in a socio-economic dilemma

**DOI:** 10.3758/s13428-023-02071-y

**Published:** 2023-02-13

**Authors:** Carlotta Cogoni, Angelica Fiuza, Leslie Hassanein, Marília Antunes, Diana Prata

**Affiliations:** 1 Instituto de Biofísica e Engenharia Biomédica, Faculdade de Ciências da Universidade de Lisboa, Campo Grande 016, 1749-016 Lisboa, Portugal; 2 Department of Health Science, Northeastern University, Boston, MA USA; 3 Department of Biological and Biomedical Sciences, University of North Carolina at Chapel Hill, Chapel Hill, NC USA; 4 Centro de Estatística e Aplicações e Departamento de Estatística e Investigação Operacional, Faculdade de Ciências, Universidade de Lisboa, Lisboa, Portugal; 5 Institute of Psychiatry, Psychology and Neuroscience, King’s College London, London, UK

**Keywords:** Prisoner’s dilemma, Social dilemma, Anthropomorphisation, Roulette, Computer, Cooperation, Competition, Social behaviour

## Abstract

In the study of human behaviour, non-social targets are often used as a control for human-to-human interactions. However, the concept of anthropomorphisation suggests that human-like qualities can be attributed to non-human objects. This can prove problematic in psychological experiments, as computers are often used as non-social targets. Here, we assessed the degree of computer anthropomorphisation in a sequential and iterated prisoner’s dilemma. Participants (*N* = 41) faced three opponents in the prisoner’s dilemma paradigm—a human, a computer, and a roulette—all represented by images presented at the commencement of each round. Cooperation choice frequencies and transition probabilities were estimated within subjects, in rounds against each opponent. We found that participants anthropomorphised the computer opponent to a high degree, while the same was not found for the roulette (i.e. no cooperation choice difference vs human opponents; *p* = .99). The difference in participants’ behaviour towards the computer vs the roulette was further potentiated by the precedent roulette round, in terms of both cooperation choice (61%, *p =* .007) and cooperation probability after reciprocated defection (79%, *p* = .007). This suggests that there could be a considerable anthropomorphisation bias towards computer opponents in social games, even for those without a human-like appearance. Conversely, a roulette may be a preferable non-social control when the opponent’s abilities are not explicit or familiar.

**Supplementary Information:**

The online version contains supplementary material available at 10.3758/s13428-023-02071-y.

## Introduction

Anthropomorphisation is the tendency to attribute human-like characteristics to non-human agents in order to rationalise their behaviour (Epley et al., 2007). While ‘human-likeness’ has been coined as a purely physical attribute (measured from ‘very mechanical’ to ‘very human-like’, using the scale adopted by MacDorman, 2006), the concept of anthropomorphisation is more extensive than the mere perception of an agent as human-like, since it entails the combination of mind attributions (in terms of agency and experience) with perceived eeriness or familiarity. Due to the popularization of artificial intelligence in the twenty-first century, our perception of the computer is likely to have become increasingly susceptible to anthropomorphisation. A computer can be perceived as an agent with human intentions and reasoning, especially if programmed to act like a human, regardless of whether it indeed uses artificial intelligence. It is now well established that the more human-like physical features a computer displays, the greater the theory of mind process it elicits in the perceiver (Krach et al., 2008). However, the degree to which the anthropomorphisation of a computer is elicited *without* manipulation of its physical features remains to be researched. At the same time, computer anthropomorphisation seems to be dependent on an individual’s a priori tendency to anthropomorphise other objects (de Kleijn et al., 2019). As a result, while computers may have had an appropriate role as non-social control conditions in psychological experiments some decades ago, the assumption that computers are not perceived to possess social features may not hold true in current times and should be questioned. This has important implications for the design of social psychology experiments, as well as for the design of novel human–computer interface devices.

**Fig. 1:**
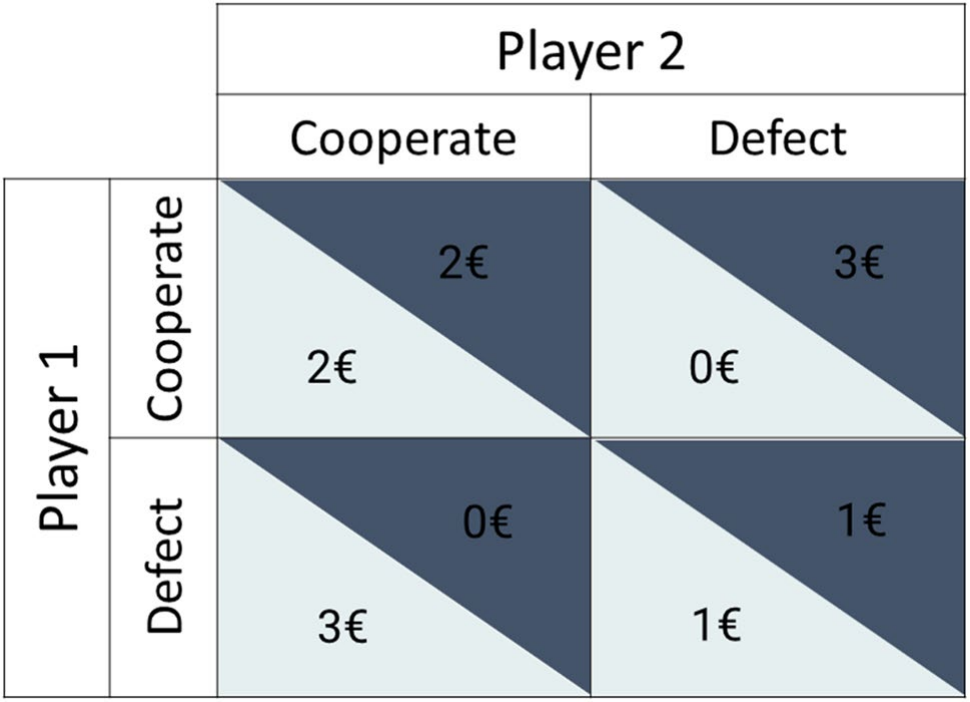
Prisoner’s dilemma payoff matrix. Each cell of the payoff matrix corresponds to a different outcome of a social interaction: both player 1 and player 2 choosing to cooperate (CC) pays €2 to both players; player 1 cooperating and player 2 defecting (CD) pays €0 to player 1 and €3 to player 2; player 1 defecting and player 2 cooperating (DC) pays €3 to player 1 and €0 to player 2; and both player 1 and player 2 defecting (DD) pays €1 to both players

A common psychological paradigm in which interacting with a computer has been used as a control condition for playing with a human, with the goal of studying cognitive empathy, mentalising, or theory of mind (virtually synonymous) processes (Mitchell et al., 2005), is the prisoner’s dilemma (PD) (Axelrod, 1984; Chong et al., 2007). The PD is part of a group of economic games representing theoretical models of economic behaviour (a.k.a. of game theory) (Richards & Swanger, 2006), which are used to provide insights into human socio-economic decision-making (Engemann et al., 2012). The PD game (Declerck et al., 2013; Todorov et al., 2011) involves two players who choose to either defect or cooperate, with the payoff being dependent upon their mutual choices (see payoff matrix in Fig 1, and Supplementary Material for more details on the PD paradigm).

In general, people tend to adopt social rules to interact with humans and computers in contexts that require social decisions, like economic games. Computers, therefore, are able to elicit social responses, even if at a lesser extent compared to living beings (Nass et al., 1994). However, how individuals choose to behave with a computer vs a human in the PD has not been extensively tested. It has been shown that when (male) participants played (as player 1) against a computer algorithm that mimicked human behaviour (reciprocating a defection move in 90% of the trials and cooperation in 67% of the trials), they (1) were only slightly less likely (80% vs 89%) (Rilling et al., 2012) or equally likely (Rilling et al., 2014) to cooperate with a computer compared to a human after a previous reciprocated cooperation, suggesting less loyalty to a computer player (Rilling et al., 2012), but (2) were more likely to cooperate with a computer than with a human, after a previous unreciprocated cooperation (Chen et al., 2016), suggesting less attribution of moral blame to the computer (Falk et al., 2008). Using the same data set as these studies, we also recently showed that playing against a computer (vs a human) increased the preference to unconditionally cooperate over the preference of pursuing a (more punishing) tit-for-tat strategy (whereby the subject mimics the opponent’s last move) (Neto et al., 2020), which may additionally or alternatively suggest that there is a perception that the computer is less/not capable of learning by reward or punishment (Kiesler & Waters, 1995).

Even though differences in behavioural responses towards humans and computers have been found, their full extent may go undetected due to anthropomorphisation effects, whose impact should not be neglected, in social paradigms such as the PD. As such, it is important to investigate to what extent participants believe the computer will act like a human being (i.e. it is anthropomorphised); for example, in a decreasing degree of anthropomorphisation: from being guided by an artificial intelligence algorithm, to an explicitly pre-programmed algorithm, to a random response algorithm (i.e. like a roulette). A relatively high level of anthropomorphisation is more likely to trigger feelings and intentions typical of human social interactions (Nass et al., 1994)—e.g. punishment, revenge, defence, or kindness and forgiveness—towards the computer.

We aimed to investigate, for the first time to our knowledge, the degree of anthropomorphisation towards a computer as opponent in an economic game, in terms of its automated perceived abilities, without varying the degree of any anthropomorphising physical features. We herein performed an analysis of the PD behaviour applied towards a human, a computer (as an agent putatively susceptible to anthropomorphisation), and a roulette (as an agent putatively non-susceptible to anthropomorphisation). Although the computer and roulette opponents' different susceptibility to anthropomorphisation can be theoretically assumed, the current experiment is the first to address the assumption via behavioural testing. We analysed both the participants’ subjective perception of their anthropomorphisation bias and their cooperative behaviour towards the same opponents—as explicit and implicit indications of anthropomorphisation, respectively. This was done to provide a comprehensive picture of the attitude towards a computer adversary in economic games and social dilemmas. PD response behaviour (in terms of choice frequencies and transition probabilities) was contrasted, within subjects, between the three opponent conditions to assess the extent to which a computer player was anthropomorphised. On the other hand, the play order of opponents was contrasted between subjects. The play order factor was introduced to (1) evaluate if/how an anthropomorphisation attitude towards the computer or roulette can be influenced by a previous game round with the roulette or computer, respectively (in particular, we posited that playing against a computer after a game round with a roulette might exacerbate the anthropomorphisation of the computer) and (2) to avoid confounding effects of opponent order when contrasting opponent conditions). Post-game anthropomorphisation-related subjective ratings of the opponents were also collected. We predicted that we would find (1) behaviour towards a human to be more similar to that towards a computer than to that towards a roulette; (2) that the similarity between the computer and human would be more accentuated for subjects that rated the computer with higher anthropomorphising characteristics; and (3) that this human–computer similarity was further accentuated when playing against a computer after playing against a roulette.

## Methods

### Participants

Forty-five male participants were recruited for the experiment. From these, 41 male participants between 18 and 34 years old (M = 22.96, SD = 4.52) were included in the analysis (see Supplementary Material for the exclusion description). Recruitment was conducted using the lab’s public website shared through social media, university campus posters, and word of mouth. Upon giving written informed consent and completing the experiment, participants were compensated for their time with a gift voucher card according to their gains during the games. The study was approved by a local ethics committee, in accordance with the Declaration of Helsinki (revised 1983).

A sample size of 45 participants is in line with the result of an a priori power analysis (in G*Power software) for a paired *t* test, with two tails, alpha at .05, and power at .95, using a previous study’s (Rilling et al., 2014) reported effect size of *d* = .57 (Cohen, 1988) for an effect of opponent (human vs computer) on defection choices—which indicated an estimated sample size of 43. We further note that we employed a generalised estimating equation (GEE) model with a Poisson distribution, which is a more adequate approach for the analyses of count measures compared to models that consider a normally distributed response.

### Experimental procedure

The experiment was conducted in a quiet room, lasting approximately 2 h. The experimental session started with demographic questions, followed by a training phase of 10 min in which participants received PD game instructions and faced three simulated opponents with two trials each, totalling six trials. Participants were subsequently provided with the subjective rating questionnaires: Mind Attribution [from not at all (1) to extremely (7) (Gray et al., 2007); Rating for Human Likeness (from very mechanical (1) to very human-like (9)], Familiarity (from very strange (1) to very familiar (9)]; and Eeriness [from not eerie (0) to extremely eerie (10)] (MacDorman, 2006). More details are available as Supplementary Material.

### Task paradigm and study design

**Fig. 2:**
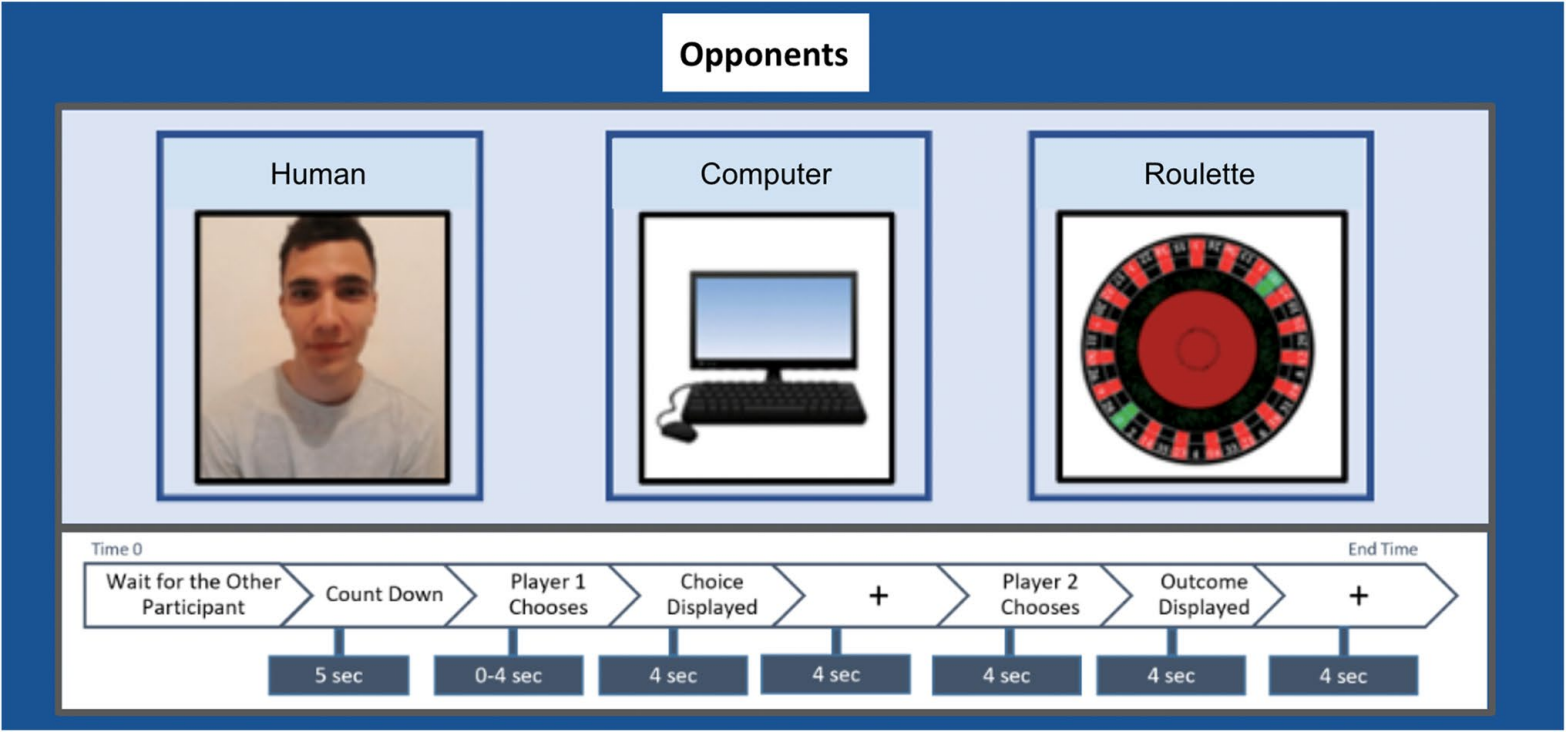
Trial timeline. Upper panel: Visual representation of the opponents: human, computer, and roulette from left to right. Lower panel: Each trial, beginning with a screen displayed for 2 s indicated the image of the opponent and the trial number. A screen of 5 s indicated waiting time for participants/opponents to be connected online, immediately followed by a countdown screen going from 5 to 0, before starting the trial. Subsequently, the participant had to decide whether to cooperate or defect in a time window of 4 s (in case of no decision, defection would be the default answer). The participant’s choice was then highlighted and displayed for 1 s to be revealed to the opponent, which then had 4 s of time to make his choice immediately after a fixation cross which was displayed for 4 s. The outcome of the trial was then displayed for 4 s. The inter-stimulus interval consisted of a last fixation cross displayed for 4 s. Trials were approximately 20 s long, one round lasted 12 min, and the three rounds had a total duration of 36 min

The experimental paradigm was divided into three different rounds of 30 trials each. All subjects played the sequential and iterated version of the PD game (see Introduction and Supplementary Material for more details and Fig. 1 for the payoff matrix). For the within-subject variable ‘opponent’, each subject competed against three opponents: a (confederate) human being, a roulette, and a computer. For the between-subject variable ‘play order’, opponents played one of two sequences: (1) human – computer – roulette (play-order HCR) or human – roulette – computer (play-order HRC). Each trial proceeded as follows. Participants were presented with images of the opponents for 2 s before each round (Fig. 2). The human opponent was always presented first to act as a baseline to then compare the degree of anthropomorphisation of the non-human opponents. All participants were led to believe that the human opponent was a real person playing against them in another room, when in reality, all three opponents responded based on the same pre-coded algorithm. Non-human opponents were therefore balanced between participants (HCR or HRC) to curb any bias that might result from the order of opponent. Participants then versed the computer and the roulette players that were represented by the images shown in Fig. 2. Such vagueness was in order not to bias participants towards a particular profile of the opponents or a priori expectations regarding the opponents’ decisions. The algorithm randomly reciprocated defection moves 90% of the time and cooperation 67% of the trials with the first opponent choice always being cooperation (Rilling et al., 2012). In addition, differently from the algorithm used in previous studies (Rilling et al., 2012), the opponent’s sequence of decisions was pre-established to guarantee a reciprocated cooperation for the first four trials minimum. The change was introduced to reduce the variability in ‘first impressions’ towards the opponents.

Participants always played as player 1 (first mover), making their choice visible to player 2 (human, roulette, or computer) before player 2 made their own choice. Figure 2 presents the timeline of the experiment and the opponents’ pictures. Additional details on the paradigm are provided as Supplementary Material.

### Statistical analysis

All analyses were performed in R version 1.3.1093. A GEE was used to fit a generalised linear model (GLM), using the *geeglm* function from package *geepack*, version 1.3-1 (Halekoh et al., 2006), to each of the dependent variables: choice frequencies (i.e. counts of cooperation [C] and defection [D] choices) and transition probabilities (i.e. counts of cooperation after each CC, CD, DC, and DD outcome), with an ‘unstructured’ correlation matrix. Given the nature of the dependent variables (i.e. counts), we considered the Poisson family of distributions to model the responses. Supplementary Material provides the models, reasons for model choices, and their estimates. Post hoc analysis consisted in testing contrasts of the factor levels using the estimated marginal means (*emmeans* function) from the *emmeans* package. Contrasts were tested in the log scale with the p-values being adjusted for multiple comparisons using the Bonferroni correction. The final results were back-transformed to their original scale for a clear and more readable interpretation.

Using the *afex* package, subjective ratings of human-likeness, familiarity, and eeriness were analysed through a mixed analysis of variance (ANOVA) with opponent as a within-subject factor and play order as a between-subject factor—with pairwise comparisons Bonferroni-corrected, and Greenhouse-Geisser corrected degrees of freedom for repeated-measures factors with more than two levels. The mind attribution questionnaire was similarly analysed, with mind dimension as the repeated measure factor having two levels: agency and experience. In addition, to understand the influence of each subjective rating on the count data, we fitted additional separate models with a three-way interaction between the rating score, the play order and opponent factors for each decision count (i.e. cooperation and defection), as well as a two-way interaction between the rating score and opponent, again adopting the GEE method. With this analysis we were able to estimate whenever, for specific values of the subjective rating scores, the rate ratio between the decision count towards one opponent vs the other (in pairwise comparisons) was significantly different from 1 (i.e. whether their confidence interval contained the value 1). These results and the data quality-driven subjects’ exclusions are fully presented as Supplementary Material. Contrasts between factor levels in both GLMs and ANOVAs were obtained using the *emmeans* package. All reported *p*-values were adjusted for multiple comparisons using the Bonferroni correction and effects with a corrected *p*-value <.05 were considered statistically significant. Plots were generated using the *ggplot2* package. The study data are available upon request to the corresponding authors. This study was not pre-registered.

## Results

### Behavioural anthropomorphisation measurements

#### Cooperation choice frequency

**Fig. 3:**
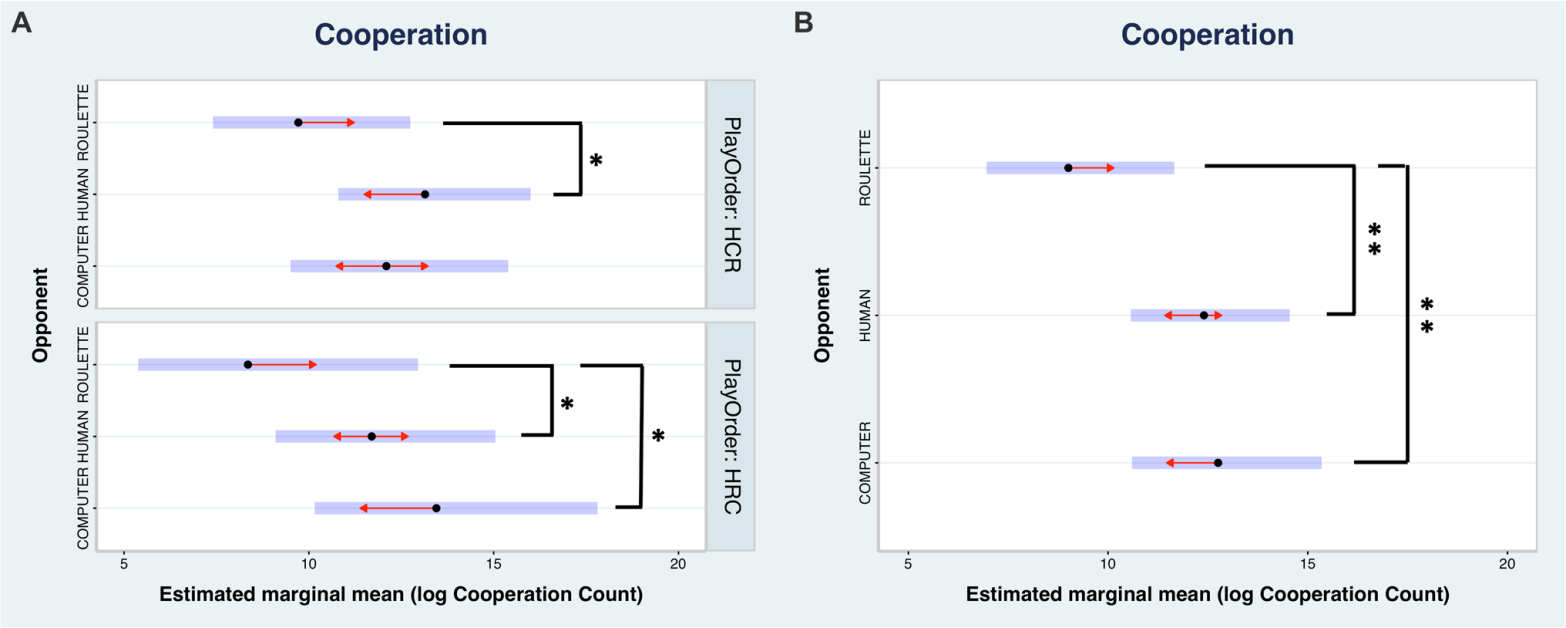
Probability of cooperation. **A** Cooperation choice as a function of opponent and play order; **B** cooperation choice as a function of opponent. The blue bars indicate the confidence intervals for the EMMs, and the red arrows indicate the comparisons among them. If the arrow from one group’s mean overlaps with the arrow from another group’s mean, their difference is not statistically significant (Bonferroni-corrected *p* > .05). Asterisks signal statistically significant effects. (**p < 0.001; * *p* < 0.05)

Cooperation choice towards the human opponent was 38% higher than towards the roulette (*p* = .001). Similarly, cooperation choice towards the computer was 41% higher than towards the roulette (*p* = .001). No significant cooperation choice differences were found between human and computer opponents (3%, *p* = .99). Cooperation choice was also similar between the two play orders (5%, *p* = .77). However, cooperation with human opponents was 35% higher than with the roulette (*p* = .03) in play-order HCR and 40% higher in play-order HRC (*p* = .02). In addition, only in play-order HRC, the cooperation choice towards the computer was 61% higher than towards the roulette (*p* = .007) (Table 1 and Fig. 3). Since results regarding defection choices are symmetrical to the above regarding cooperation as expected, they are added to the Supplementary Material, for completeness.

**Table 1:** Cooperation choice frequency estimated marginal means, and the effect of opponent on cooperation choice (pairwise comparisons) for each play order

Cooperation choice frequency					
Opponent	Play order	Rate	SE	LCL	UCL
Human	HCR	13.14	1.32	10.80	16.0
	HRC	11.70	1.50	9.10	15.1
Computer	HCR	12.10	1.49	9.50	15.4
	HRC	13.45	1.93	10.16	17.8
Roulette	HCR	9.71	1.34	7.40	12.7
	HRC	8.35	1.87	5.38	12.9
Effect of opponent on cooperation choice frequency per play order					
Opponent contrasts		Ratio	SE	*Z* ratio	*p*-value
HCR	Human vs computer	1.09	0.11	0.79	.99
	Human vs roulette	1.35	0.16	2.54	.03* (Hum > Rou)
	Computer vs roulette	1.25	0.14	1.95	.15
HRC	Human vs computer	0.87	.09	−1.33	.55
	Human vs roulette	1.40	0.17	2.72	.02* (Hum > Rou)
	Computer vs roulette	1.61	0.25	3.05	.01* (Com > Rou)
Main effect of opponent on cooperation choice frequency					
Opponent contrasts		Ratio	SE	*Z* ratio	*p*-value
Human vs computer		0.97	0.07	−0.38	.99
Human vs roulette		1.38	0.12	3.72	.001* (Hum > Rou)
Computer vs roulette		1.42	0.14	3.62	.001* (Com > Rou)

Asterisks signal statistically significant effects (Bonferroni-corrected *p* < .05), accompanied by their direction. *SE* standard error; *LCL/UCL* lower/upper confidence levels

#### Transition probability of cooperation after a cooperation-cooperation outcome

The analysis was run using data from 110 out of the 123 possible observations (i.e. number of rounds in which cooperation-cooperation outcomes occurred, among each opponent and each subject). The probability of cooperation after a CC outcome was similar among opponents (<5%, *p*s > .77), and between the two play orders (.004%, *p* = .97). No pairwise comparisons between opponents in each play order showed significance below *p* = .99, (<8%) (Supplementary Table S8).

#### Transition probability of cooperation after a cooperation-defection outcome

The analysis was run using data from 67 out of the 123 possible observations (i.e. number of rounds in which cooperation-defection outcomes occurred, among each opponent and each subject). Probabilities of cooperation after a CD outcome were similar among opponents (<27%, *p*s > .17) and they did not significantly differ between the two play order sequences (22%, *p = .*17). No pairwise comparisons between opponents in each play order showed significance below *p = .*17 (<48%) (Supplementary Table S9).

#### Transition probability of cooperation after a defection-cooperation outcome

The analysis was run using data from 61 out of the 123 possible observations (i.e. number of rounds in which defection-cooperation outcomes occurred, among each opponent and each subject). Probabilities of cooperation after a DC outcome were similar among the opponents (<7%, *p*s = .99) and between the two play order sequences (8%, *p = .*55). No pairwise comparisons between opponents in each play order showed significance below *p = .*10 (<34%) (Table S10).

#### Transition probability of a cooperation after a defection-defection outcome

The analysis was run using data from 89 out of the 123 possible observations (i.e. number of rounds in which defection- defection outcomes occurred, among each opponent and each subject). Probabilities of cooperation after a DD outcome were similar among opponents (<30%, *p*s <*.*28) and between the two play order sequences (8%, *p = .*60). However, (only) in play-order HRC the probability of cooperation after a DD outcome with computer opponents was 79% higher than with the roulette opponents (*p* = .007), while no difference was found between human and computer (29%, *p = .*22) or between human and roulette (28%, *p = .*64). No pairwise comparisons between opponents in play-order HCR showed significance below *p = .*60 (<40%) (Table 2).

**Table 2:** Probability of cooperation after a defection-defection (DD) outcome estimated marginal means (EMMs), and effect of opponent on the probability of a cooperation after a DD outcome (pairwise comparisons), in each play order

Transition probability of a cooperation after DD					
Opponent	Play order	Rate	SE	LCL	UCL
Human	HCR	3.70	0.55	2.76	4.96
	HRC	3.19	0.52	2.32	4.38
Computer	HCR	2.63	0.64	1.64	4.22
	HRC	4.46	0.65	3.36	5.92
Roulette	HCR	2.78	0.57	1.87	4.15
	HRC	2.49	0.43	1.78	3.48
Effect of opponent on transition probability of a cooperation after DD per play order sequence					
Opponent contrasts		Ratio	SE	*Z* ratio	*p*-value
HCR	Human vs computer	1.40	0.37	1.29	.60
	Human vs roulette	1.33	0.33	1.15	.75
	Computer vs roulette	0.95	0.29	−0.18	.99
HRC	Human vs computer	0.71	0.14	−1.78	.22
	Human vs roulette	1.28	0.26	1.24	.64
	Computer vs roulette	1.79	0.34	3.04	.007*(Com > Rou)
Main effect of opponent on transition probability of cooperative choice after DD					
Opponent contrasts		Ratio	SE	*Z* ratio	*p*-value
Human vs computer		1.0	0.16	0.01	.99
Human vs roulette		1.3	0.21	1.68	.28
Computer vs roulette		1.3	0.234	1.46	.44

Asterisks signal statistically significant effects (Bonferroni-corrected *p* < .05), accompanied by their direction. *SE* standard error; *LCL/UCL* lower/upper confidence levels

### Subjective anthropomorphisation ratings

#### Mind attribution questionnaire

**Fig. 4:**
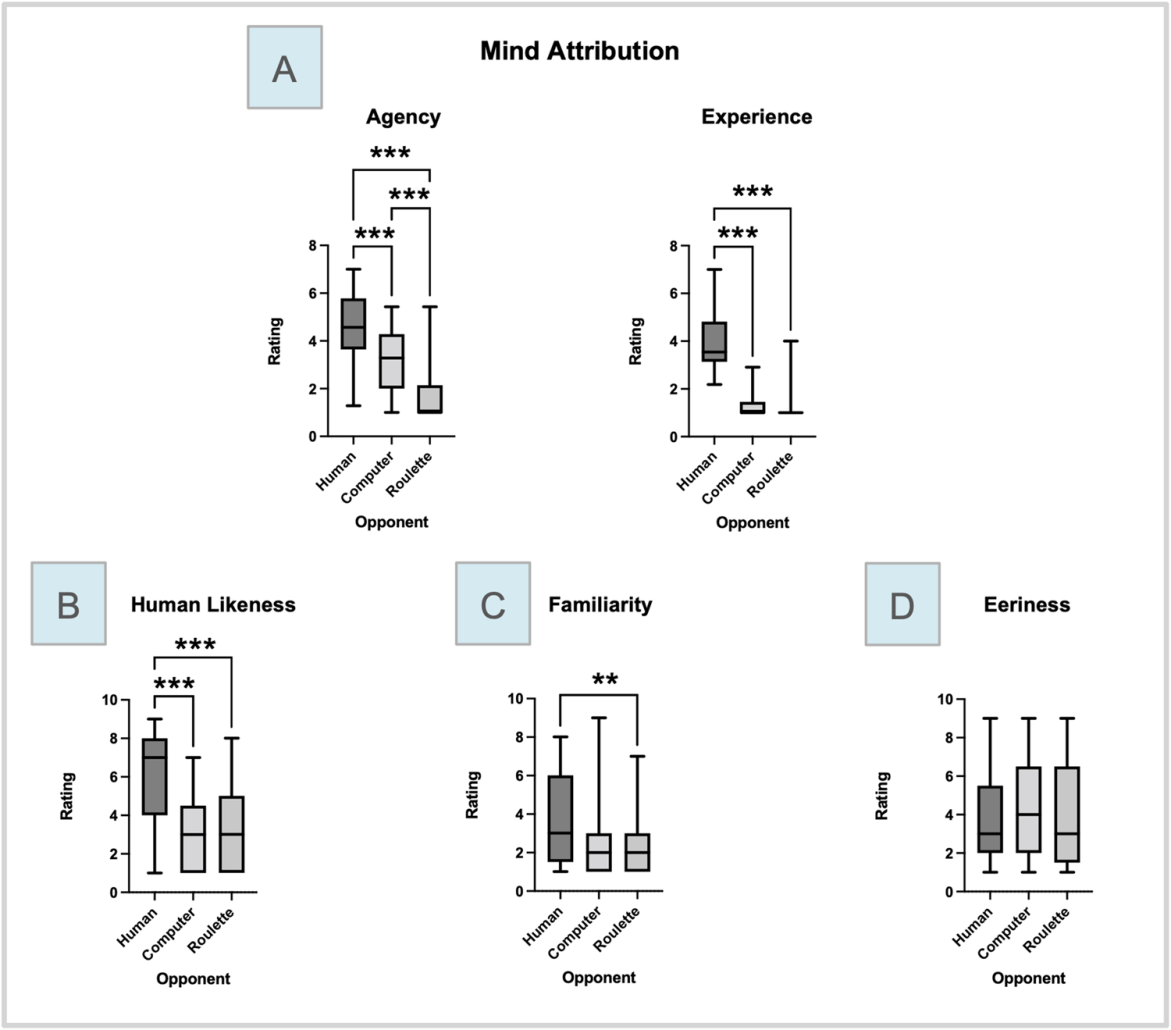
Subjective anthropomorphisation ratings. **A** Mind attribution; **B** human-likeness; **C** familiarity; **D** eeriness. Scores are represented by box-and-whisker plot, with central lines indicating the mean participant rating of each opponent. Asterisks signal statistically significant effects. (***p < .0001; ** *p* < .001)

We found a main effect of opponent on mind attribution ratings (*F*(1.46, 57) = 98.09, *p* < .001, η^2^p = 0.72), with the human receiving higher mind attribution than both the computer (*p* < .0001) and the roulette (*p* < .0001), and the mind attribution towards the roulette was significantly lower than that towards the computer (*p* < .003). A main effect of mind dimension on mind attribution was also found (*F*(1, 39) = 84.52, *p* < .001, η^2^p = 0.68), indicating that regardless of the opponent partner or the play order, agency was generally perceived as higher than experience (*p* = .026). An interaction between mind dimension and opponent on mind attribution was also found (*F*(1.90, 73.97) = 27.72, *p* < .001, η^2^p = 0.42), showing that while agency attribution was equidistantly different between the three opponents (human > computer > roulette; each of the three pairwise comparisons—i.e. human vs computer, human vs roulette, and computer vs roulette—yielding *p* < .0001), experience attribution was four times higher in the human than both the computer opponent (*p* < .0001) and the roulette opponent (*p* < .0001)—which were identical themselves (*p = .*999) (Table 3). None of the other main effects or interactions were statistically significant (*p > .*14) (Fig. 4).

**Table 3:** Mind attribution, human-likeness, familiarity, and eerieness ratings regarding the opponents

Subjective ratings		Opponent	Mean	SE	LCL	UCL
Mind attribution	Agency	Human	4.64	0.18	4.29	4.99
		Computer	3.20	0.18	2.84	3.55
		Roulette	1.72	0.18	1.37	2.07
	Experience	Human	4.12	0.18	3.77	4.47
		Computer	1.24	0.18	0.89	1.59
		Roulette	1.23	0.18	0.88	1.58
Human-likeness		Human	6.10	0.32	5.46	6.74
		Computer	2.98	0.32	2.34	3.61
		Roulette	2.95	0.32	2.31	3.58
Familiarity		Human	3.53	0.31	2.91	4.16
		Computer	2.54	0.32	1.91	3.16
		Roulette	2.25	0.32	1.62	2.87
Eeriness		Human	3.84	0.41	3.02	4.66
		Computer	4.37	0.41	3.54	5.19
		Roulette	4.02	0.41	3.19	4.84
Main effect of opponent on mind attribution ratings						
Opponent contrasts		*t*	DF	*d*	*p*-value	
Human vs computer		10.04	78	1.86	.0001* (Hum > Com)	
Human vs roulette		13.48	78	2.50	.0001* (Hum > Rou)	
Computer vs roulette		3.44	78	0.64	.003* (Com > Rou)	
Main effect of mind dimension on mind attribution ratings						
Mind dimension		*t*	DF	*d*	*p*-value	
Agency vs experience		2.3	38	2.94	.026* (agency > experience)	
Interaction between mind dimension and opponent						
Type of attribution		Opponent contrast	*t*	DF	*d*	*p*-value
Agency		Human vs computer	5.95	118	1.10	<.0001* (hum > com)
		Human vs roulette	12.01	118	2.21	<.0001* (hum > rou)
		Computer vs roulette	6.07	118	1.12	<.0001* (com > rou)
Experience		Human vs computer	11.85	118	2.18	<.0001* (hum > com)
		Human vs roulette	11.88	118	2.19	<.0001 * (hum > rou)
		Computer vs roulette	0.03	118	0.01	.99
Main effect of opponent on human-likeness ratings						
Opponent contrasts		*t*	DF	*d*	*p*-value	
Human vs computer		8.33	78	1.89	<.0001* (Hum > Com)	
Human vs roulette		8.41	78	1.90	<.0001* (Hum > Rou)	
Computer vs roulette		0.08	78	0.02	.99	
Main effect of opponent on familiarity ratings						
Opponent contrasts		*t*	DF	*d*	*p*-value	
Human vs computer		2.38	78	0.54	.06 (Hum > Com)	
Human vs roulette		3.07	78	0.69	.009* (Hum > Rou)	
Computer vs roulette		0.08	78	0.16	.99	

Asterisks signal statistically significant effects (Bonferroni-corrected *p* < .05), accompanied by their direction. *SE* standard error; *LCL/UCL* lower/upper confidence levels

#### Human-likeness

We found a main effect of opponent (*F*(1.43, 55.66) = 46.73, *p* < .001, η^2^p = .55) on human-likeness ratings, with the human opponent being perceived as more human-like than the computer (*p* < .0001) and more human-like than the roulette (*p* < .0001). On the contrary, the computer and roulette were perceived as similarly human-like *(p* = .999). All other main effects and interactions were not statistically significant (*p*s *> .*52) (Fig. 4).

#### Familiarity

We found a main effect of opponent on familiarity ratings (*F*(1.92, 74.77) = 5.18, *p* = .009, η^2^p = .12), with the human opponent being perceived as more familiar than the roulette (*p* = .009) and the computer, even though the difference was only marginally significant (*p* = .06). On the contrary, the computer and roulette were perceived as similarly familiar (*p* = .999). All the other main effects or interactions were not statistically significant (*p*s > .67) (Fig. 4).

#### Eerieness

Human, computer, and roulette opponents were perceived as equally not eerie, as indicated by the absence of a main effect or interaction (*p*s *> .*45) (Fig. 4).

### Influence of subjective ratings on choice frequencies.

**Fig. 5:**
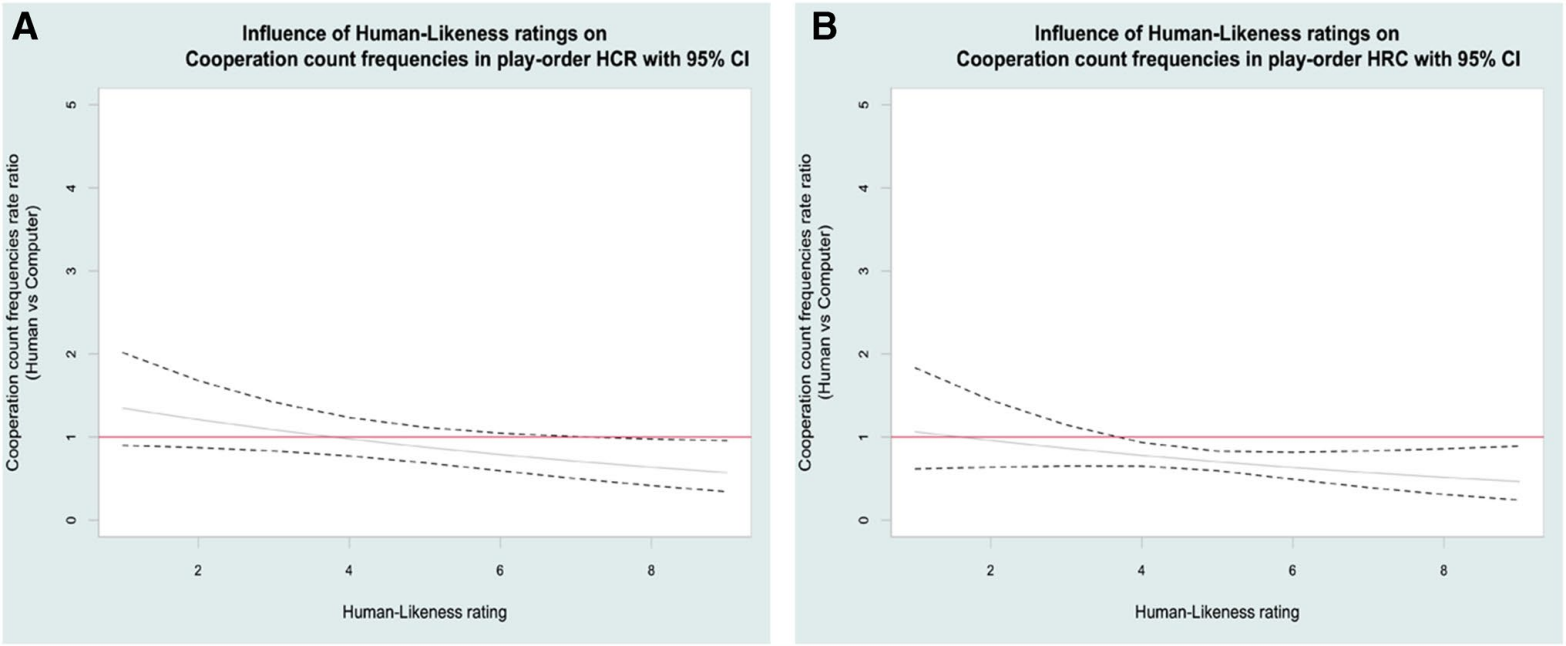
Influence of human-likeness ratings on cooperation count frequencies. **A** Play-order HCR; **B** Play-order HRC. The red line indicates a rate ratio equal to 1, with no difference between the cooperation count of the human and computer opponents. The dashed line indicates the rate ratio 95% CI upper and lower boundaries. The rate ratio is considered significantly different when at a certain value of the human-likeness ratings, their 95% CI upper and lower boundaries do not contain the value 1. A significant rate ratio smaller than 1 indicates a cooperation count towards the computer opponent higher than towards the human opponent

For specific values of the subjective rating scores, we estimated if the rate ratio between the decision count towards one opponent vs the other (in pairwise comparisons) was significantly different from 1 (i.e. whether their confidence interval contains the value 1). Only participants that later attributed the *highest* human-likeness rating to the computer and the human (even though the human opponent was on average perceived as more human-like than the computer), cooperated more with the computer than with the human (*M*_*human-likeness rating range*_ = 8–9; 95% CI _*rate-ratio*_ [LCL_range_: 0.34–0.42; UCL_range_: 0.96–0.98]). This only happened (at the highest human-likeness rating) when the computer opponent play was immediately preceded by the human opponent play (Fig. 5). When the computer was preceded by the roulette, participants who later attributed both *highest* or just *medium-to-high* human-likeness to the computer and the human, cooperated more with the computer than with the human (*M*_*human-likeness rating range*_ = 4–9; 95% CI _*rate-ratio*_ [LCL_range_: 0.24–0.65; UCL_range_: 0.89–0.93]) (Fig. 5). Similar computer and roulette human-likeness attribution ratings were also indicative of a higher cooperation towards the computer vs the roulette in both play orders.

In addition, the different decision choices towards computer and roulette became evident in the case of mind attributions but only when the computer opponent is preceded by the roulette one. In case of *low* experience attribution to computer and human opponents, the defection towards the computer was higher than towards the human (*M*_*experience rating range*_ = 2.18–3.91; 95% CI _*rate-ratio*_ [LCL_range_: 0.35–0.60; UCL_range_: 0.93–0.99]). At the *lowest* experience attribution to computer and human opponents, the defection pattern is reversed (i.e. the defection towards the computer is lower than towards the human), (*M*_*experience rating range*_ = 1.18–1.91; 95% CI _*rate-ratio*_ [LCL_range_: 1.001 – 1.01; UCL_range_: 1.78 – 1.94]). Only selected findings are summarised above; for detailed results, see the Supplemental Online Material including Attachments 1 and 2).

## Discussion

In experimental psychology, computers are assumed to represent non-social or non-human agents and thus are often used in control conditions of social/human conditions in experimental paradigms (Chen et al., 2017; Neto et al., 2020; Rilling et al., 2012, 2018). Given the absence of support for the later premise, we designed the present study in order to challenge it. Besides an inquiry on subjective anthropomorphising attitude (i.e. explicit anthropomorphisation), we tested whether we could detect anthropomorphising behaviour (i.e. implicit anthropomorphisation*)* towards computer opponents in an economic game, by contrasting it with that towards humans and roulettes. Additionally, we utilised different opponent play order sequences, and no manipulation of its physical characteristics towards a human-like figure (which is already known to increase anthropomorphisation; Krach et al., 2008). [We note that while ‘human-likeness’ has been coined as a purely physical attribute, measured from ‘very mechanical’ to ‘very human-like’, using the scale adopted by MacDorman, 2006, the concept of anthropomorphisation is more extensive than the mere perception of an agent as human-like, as it entails the combination of mind attributions—in terms of agency and experience—with perceived eeriness or familiarity]. We hope our findings will help inform the most appropriate choice of control condition (computer or roulette) in future neuroeconomic experimental paradigms and improve interpretation of existing literature.

In summary (and in detail below), we found that behaviour towards a human opponent almost always differed from that towards a roulette. On the contrary, the behaviour towards the computer opponent differed from that towards a roulette only when considering total cooperative choices and in the case of an immediate previous reciprocated defection. In both cases, the computer anthropomorphisation, as indicated by a higher cooperation behaviour, occurred when playing with a roulette preceded playing with a computer. This suggests people might tend to be forgiving and more readily create the basis for trust and cooperation when they are dealing with human beings, but also with computers (i.e. showing anthropomorphisation), especially when they are contrasted with a roulette via a recent interaction.

### Similarity in cooperation towards computers and humans, and dissimilarity towards roulettes, may reflect anthropomorphisation

We found that behaviour, in terms of PD player 1 cooperative choices, towards a computer was not significantly different than that towards a human opponent (Table 1). This result challenges the adequacy of computers as controls to human conditions, at least in a setting where no prior information on the attributes or modus operandi of the computer is given to the study participant. It may indicate that the (commonly assumed) non-social attributes of a computer are not sufficiently perceived by study participants to make them behave significantly differently towards a computer versus a human in a social decision-making paradigm. On the other hand, behaviour towards a roulette was significantly different than that towards a human. With roulettes, subjects mostly adopted the strategy of defecting (16% more than with the human) to gain at least €1 in each trial and ensure a minimum gain. This exemplifies the maximum-gain strategy (leading to Nash equilibrium) which occurs in single-shot (i.e. non-iterative) versions of the PD. This suggests that the roulette—unlike the computer—was not perceived as able to account for the subject’s previous choices in the game (i.e. learn). In other words, whilst the roulette was not anthropomorphised, the computer was.

### Anthropomorphisation of a computer may be augmented by recent interaction with roulette

Moreover, we observed that total cooperative behaviour (i.e. probability to cooperate across the whole game round) was profoundly affected by the computer vs roulette chronological play order in the same session (Table 1). Specifically, cooperative behaviour towards the computer opponent was higher (61%) compared to that towards the roulette only when the subject had played against the roulette beforehand (i.e. HRC play-order); there was no difference in the reverse play order (i.e. HCR). In other words, playing with a roulette beforehand seems to increase the degree of anthropomorphisation towards a computer in a subsequent game.

### Anthropomorphisation of a computer is not affected by a previous outcome, except that of a reciprocated defection

When we tested the effect of opponent and of play order on the probability of cooperating after a specific trial outcome, the degree of anthropomorphisation depended on the type of outcome. When a participant’s cooperation was reciprocated (CC), the probability of cooperation in the following trial was similarly high for all opponents (Table S8). Likewise, when the participant’s cooperation was not reciprocated (CD), the probability of cooperation in the following trial was similarly low for either opponent (Table S9). This indicates the predominance of a tit-for-tat strategy (Neto et al., 2020). Therefore, after these two outcome cases (CC and CD), either a roulette is also being anthropomorphised (which we think is unlikely) or evidence of anthropomorphisation (i.e. a higher similarity between computer and human opponent treatment than between human and roulette) is not detectable. Either way, any existing anthropomorphisation of the opponent did not significantly influence choice after an outcome of cooperation reciprocation or cooperation betrayal.

When participants had adopted a defensive behaviour (i.e. a defection choice) and they were surprised by a cooperation choice by the opponent (i.e. DC), the probability of cooperation in the following trial was again equal for each opponent (Table S10). However, when the defective behaviour of the participant was reciprocated with a defection (DD), the probability of cooperation in the following trial with the computer opponent was higher (79%) than with the roulette opponent (Table 2), but only when playing with a computer occurred immediately after playing with a roulette (and not when it was preceded by playing with a human, i.e. HCR play-order). This also partially supports the presence of anthropomorphisation of the computer opponent, and that it is augmented by a previous interaction with a roulette—‘partially’ because behaviour with a human opponent was intermediate; it was not significantly different from that towards the computer or roulette.

### Behavioural anthropomorphisation is partially reflected in mind attribution subjective ratings

As we expected, mirroring the signs of anthropomorphisation in the behavioural results, participants attributed the human opponent the highest perceived agency, followed by the computer and then the roulette (the opponent accounted for 79% of the variance in mind attribution ratings unexplained by the mind dimension or the play order—a large effect size). Also, the agency scores of the three opponents were higher than their experience scores, indicating that all opponents were seen more as agents (i.e. deliberately making choices in the PD game) than as beings that experience emotions (Gray et al., 2007; Gray et al., 2011) (the mind dimension explained 68% of the variance in mind attribution ratings, left unexplained by play order or opponent—a large effect size). However, the computer was perceived as no different from the roulette in terms of experience attribution or human-likeness or familiarity, to our participants. We believe that this may be because when participants are requested to rate an inanimate agent (i.e. the computer or the roulette), they reply rationally counteracting the implicit perception occurred while playing the game. That is, in order to comply with common knowledge, participants might be lured to rationally describe what a computer and a roulette are reasonably capable of. Likely as a consequence, our analysis of the influence of subjective ratings on cooperation and defection choices could not fully explain the anthropomorphisation process detected during the PD. Nevertheless, some interesting hints have emerged, as discussed next.

As expected, the human opponent was rated with the highest human-likeness attribution. However, in the rare case of *similar* high human-likeness attribution to human and computer, and where computer play was preceded by human play, the cooperation towards the computer was in fact significantly higher (36% to 43%) than towards the human. On the contrary, when the computer game was preceded by the roulette, the computer anthropomorphisation dramatically increased to the extent that not only at *high* but also at *medium-to-high* human-likeness attribution the cooperation towards the computer significantly surpassed (22% to 54%) cooperation towards the human. These results confirm the importance of play order, where a previous interaction with a roulette before the computer augments the computer’s anthropomorphisation.

As an additional indication of the importance of the play order in influencing the opponent subjective perceptions, when computer and human opponents were rated with *low* experience the defection towards the computer was significantly higher (23% to 41%) than towards the human (HCR play-order). This suggests—in an intuitively predictable manner—that when the computer is perceived as scarcely able to experience emotions the defective behaviour towards it increases, as compared to the human. Conversely, when the computer opponent is preceded by the roulette one, and computer and human opponents are rated with the *lowest* experience, subjects defected significantly less (34% to 39%) with the computer than with the human. This confirms a predominant anthropomorphisation process of the computer opponent. The above subjective rating findings suggest that there are anthropomorphising attitudes which may even lead subjects to surpass towards computers the cooperation degree they show towards humans. This is not unprecedented given a previous report of higher probability of cooperation after a previous cooperation-defection outcome with computer vs human (Chen et al., 2016). However, it may be confounded by the human always being the first opponent, and therefore the one with which participants were still adapting to the game and still ‘perfecting’ their strategy.

### Choosing the best non-social control condition in socio-economic dilemmas

Once the validity of the computer opponent in economic games as a non-social control is questioned, the specific comparison between computer and roulette opponents, in the present study, may represent a step forward in social economics research. We believe that the use of the roulette as an independent opponent has been overlooked, leaving a gap in the available data on the exploration of its advantages compared to adopting a computer for this purpose. In one study, a roulette was used in the multiplayer game ‘Take Some’, an alternative version of the PD, with the only purpose of identifying a cut-off number or threshold (Guyer et al., 1973). Alternatively, roulette conditions have been used with the aim of controlling the response to monetary reinforcement, independent of any social interaction. Importantly, these control trials were designed as intrinsically different than the experimental trials against either human or computer opponents, preventing any comparisons with the latter (see Rilling et al., 2004; Sanfey et al., 2003, for further details of these control trials).

One solution for ensuring an adequate social control condition may be using a computer opponent condition where clear statements decreasing/limiting its agency degree would be provided, thus making them more suitable as non-social controls when confronted with human opponents. For example, it could be explained at the beginning of the economic game whether the computer follows an algorithm or mimics a roulette in its actions, without the need to add a roulette opponent to the design. Future studies should verify whether the present results can be modified by providing such brief a priori information.

There is one caveat regarding our study design, which may limit its comparability with the aforementioned studies (Neto et al., 2020; Rilling et al., 2012). In those studies, participants met with human actors, i.e. confederates (matched for age and sex), before engaging in the PD paradigm. The act of engaging with the human opponent in the flesh may have strengthened the belief that an opponent will indeed be human, further personifying and differentiating the ‘human’ opponent from the computer. In contrast, in our study we told participants they would play with three different players from another room in the facility without providing additional details, apart from a photo of each. This was done to avoid noise arising from the variability with which the confederate would present himself day-to-day (regarding mood, politeness, clothing appearance, etc.).

### Statistical approach to the prisoner’s dilemma data analysis

Although not the main goal of the present study, we herein provided new insight into the statistical analysis of PD data by suggesting what we believe to be a statistically more appropriate approach than those used in previous work. Because of the constraints of the algorithm which reciprocates cooperation 67% of the time and defection 90%, the outcomes frequencies (i.e. CC, CD, DC, and DD) do not have equal occurrence. Hence, comparing outcome frequencies in an ANOVA, as commonly done in literature, is not the most appropriate statistical analysis choice. Both frequencies of cooperation and defection choices and the frequency of cooperation after each of the four possible outcomes are indeed ‘count’ data and, as such, need to be analysed with Poisson distributions. A normal distribution might be a fair approximation to a Poisson one only for data with a higher number of observations (i.e. above 30) than most behavioural studies collect.

## Conclusion

The present research suggests special care in the design of non-human opponents as control conditions for human ones in socio-economic games, due to a potentially high anthropomorphising tendency towards computers by study participants. This pattern may be counteracted by providing prior details on the computer characteristics, leading to a change in mind attribution and consequently in the subject’s behavioural responses during the game. Future studies should be extended to the female population to verify whether men and women show different levels of anthropomorphisation of non-social opponents in economic games.

### Supplementary information

ESM 1 (DOCX 1703 kb)
