# Supplementary material for: Computer anthropomorphisation in a socio-economic dilemma
Source: Behav Res Methods. 2023 Feb 13;56(2):667–79. doi: 10.3758/s13428-023-02071-y (PMC10830593; doi:10.3758/s13428-023-02071-y)
Supplement: Supplementary file 1 — (DOCX 1703 kb) [file 13428_2023_2071_MOESM1_ESM.docx]

**Supplementary Material**

**Introduction**

In the game’s *sequential* version, PD players have the possibility to make their choice one after the other (i.e. Player 1 chooses and Player 2 is then able to view Player 1’s choice before making his/her own choice). If the version is also *iterated*, players are asked to play several times with the same opponent during one game session – in contrast with the *single-shot* version (Dijkstra & van Assen, 2017). In the single-shot version of the game, the most profitable strategy is to always defect (i.e.‘unconditional defection’), leading to Nash equilibrium (Osborne & Rubinstein, 1999). However, in the sequential and iterated PD, people tend to adopt a relatively more cooperative attitude (Bó & Fréchette, 2011). In this version, mutual cooperation provides the maximum gain for both players (see (Declerck et al., 2013) for reasons that induce people to cooperate instead of defecting during economic games). However, since any agreement or previous personal relationship before the game is forbidden, the risk that the other player is tempted to increase his\her gain by defecting is not negligible. This risk makes mutual cooperation also an extremely unstable strategy. As such, people tend to make their choices depend on the opponent’s previous choices. For a Player 1 who cooperates, receiving a cooperative choice from the opponent is thought to trigger feelings of friendship, trust, gratitude and an intension to reciprocate with further cooperation. Conversely, receiving a defection may trigger feelings of rejection, indignation, unfairness, and an intension not to cooperate in the next move for one’s own protection, and/or punishment of the opponent.

**Methods**

**Participants**

Forty-one male participants between 18 and 34 years old (M = 22.96, SD = 4.52) were included. All subjects gave written informed consent, and the study was approved by a local Ethics Committee, in accordance with the Declaration of Helsinki (revised 1983). Individuals participating in this study were recruited using the lab’s public recruitment website divulged via social media, university campus posters and word of mouth. The experiment was conducted in a quiet room and participation lasted approximately 2 hours. Participants were compensated for their time with a gift voucher card according to their gains during the games.

**Experimental procedure**

Participants were under the guise that they were competing against other players located in other rooms of the building, whilst in reality there were no real human opponents, and all opponent choices were controlled by an identical pre-programmed computer algorithm. To reinforce this cover story, a photo of each participant was taken with the false belief that it would be shown to his opponents during the game. The experiment was run on a laptop (15.6'' screen); MATLAB version 8.3.0 (R2014a) with Psychtoolbox3 software was used for stimulus presentation and Qualtrics for online administration of the subjective rating questionnaires. The experimental session started with demographic questions, followed by a training phase of 10 minutes in which participants received PD game instructions and faced 3 simulated opponents with two trials each, totaling 6 trials. No money was earned in the training session, and a training quiz was subsequently administered to assure that the instructions were completely clear. If any question was answered incorrectly, participants were verbally instructed on why their answer choice was incorrect, and why another answer was correct. If necessary, subjects repeated the tutorial. Participants were subsequently provided with the subjective rating questionnaires: Mind Attribution (from not at all (1) to Extremely (7) (Gray et al., 2007); Rating for Human Likeness (from very mechanical (1) to very human-like (9)), Familiarity (from a very strange (1) to very familiar (9)), and Eeriness (from not eerie (0) to extremely eerie (10)) (MacDorman, 2006).

**Task paradigm and study design**

The experimental paradigm was divided in 3 different rounds of 30 trials each. All subjects played the sequential and iterated version of the Prisoner Dilemma game (see Introduction for more details and Figure 1 for the payoff matrix).

As the within-subject variable ‘Opponent’, each subject competed against three opponents: a (confederate) human being, a roulette, and a computer. As the between-subject variable ‘Play-order’, opponents played one of 2 sequences: 1) human – computer – roulette (Play-order HCR) or human – roulette – computer (Play-order HRC). Each trial proceeded as follows. Participants always played as Player 1 (first mover), making their choice visible to Player 2 (Human, Roulette, or Computer) before Player 2 made their own choice. After instructions, participants were first presented with the visual image of the opponent and the indication of the trial number, then a screen indicating to “wait for the other participants”, a countdown screen immediately followed, counting from 5 to 0 before the start of the trial. Player choices were made by pressing the up and down keys highlighted on the keyboard. See Figure 2 for the timeline of the experiment.

In the final screen, the amount of money won by the participant was displayed as a proportion of the three rounds gains sum. Participants could earn 0€, 5€ or 10€ according to performance (< 72€, ≥ 72€ < 119€, and ≥ 119€ respectively), on top of the 5€ baseline they received for participating in the study regardless of the game outcome. The algorithm controlling the opponent’s choices is a modified version of one previously used which randomly reciprocated defection moves 90% of the time and cooperation 67% of the trials with the first opponent choice always being cooperation (Rilling et al., 2012), with the difference that the opponent’s sequence of decisions was pre-established and guaranteed a reciprocated cooperation for the first 4 trials minimum. The change was introduced to reduce the variability of “first-impressions” toward the opponents. In addition, to reduce variability in terms of perceived familiarity of the human opponent, its name was selected from a list of the 10 most frequent Portuguese male names.

**Statistical analysis**

All analyses were performed in R version 1.3.1093. The Generalized Estimating Equation (GEE) approach was used to fit a generalized linear model (GLM) to each of the dependent variables: choice frequencies (i.e., counts of cooperation and defection choices) and transition probabilities (i.e., counts of cooperation after each CC, CD, DC and DD outcome). As suggested in Hanley and colleagues (Hanley et al., 2003), a GEE approach was adopted to model the multiple-event data while accounting for the dependency of the observations within each participant (i.e., the within subject factor “opponents”). The method allows to obtain robust standard errors and confidence intervals estimates for the model parameters, despite the existence of multiple observations per participant. This is achieved by considering a correlation structure to adjust for the dependency of within-participants observations (Hanley et al., 2003). We considered a correlation matrix of the type “unstructured” in order to allow correlations to differ across opponents. Given the nature of the dependent variables (i.e., counts), we considered the Poisson family of distributions to model the responses (See Supplementary material for the models and their estimates). The models were fitted using the *geeglm* function from package *geepack*, version 1.3-1 (Halekoh et al., 2006). Post-hoc analysis consisted in testing contrasts of the factors’ levels using the estimated marginal means (*emmeans* function) from *emmeans* package. Since the use of the Poisson family to model the data requires the use of the logarithmic link function, the contrasts were tested in the log scale with the p-values being adjusted for multiple comparisons using the Bonferroni correction. The final results were back-transformed to their original scale for a clear and more readable interpretation.

Using the *afex* package, subjective ratings of human-likeness, familiarity and eeriness were analyzed through a mixed ANOVA with opponent as within-subject factor and play-order as a between-subject factor – with pairwise comparisons Bonferroni-corrected and Greenhouse-Geisser corrected degrees of freedom for repeated-measures factors with more than two levels. The mind attribution questionnaire was similarly analyzed, with mind dimension as the repeated measure factor having 2 levels: agency and experience. In addition, to understand the influence of each subjective rating on the counts data, we fitted additional separate models with a 3-way interaction between the rating score, the play-order and opponent factors for each decision count (i.e., cooperation and defection), as well as a 2-way interaction between the rating score and opponent, again adopting the GEE method. With this analysis we were able to estimate whenever, for specific values of the subjective rating scores, the rate ratio between the decision count toward one opponent vs the other (in pairwise comparisons) was significantly different from 1 (i.e., if their confidence interval contains or not the value 1). A rate ratio of 1 indicates that there is no difference between one opponent and the other (in pairwise comparisons) at specific values of subjective rating (in which case confidence intervals of the rate ratio contain 1). These results are fully presented as supplementary material.

Contrasts among factor levels in both GLM models and ANOVAs were obtained using the *emmeans* package. All reported p-values were adjusted for multiple comparisons using the Bonferroni correction and effects with a corrected p-value < .05 were considered statistically significant. Plots were generated using the *ggplot2* package. Data from 4 participants were not included in the analysis – one participant was excluded due to lack of attention during the experiment, and 3 participants were excluded due to prior knowledge on the PD game which undermined the belief of the human opponent’s existence. Therefore, the final analyzed sample comprised 41 participants. The study’s data are available upon request to the corresponding authors. This study was not preregistered.

**Results**

**Choice frequencies and transition probabilities’ Gee models coefficient’s.**

In the following tables the parameters estimated from the gee models are reported. In each model a Poisson distribution and an unstructured correlation matrix was specified. The Opponents are indicated as follow: Opponent 1= HUM, Opponent 2= CPU, Opponent 3= ROU. The play-orders are indicated as follow: PlayOrder_1CR_2RC 1= HCR, PlayOrder_1CR_2RC 2= HRC.

Table S1. Gee model on cooperation choices counts

| geeglm(Count~PlayOrder_1CR_2RC * Opponent,data=Subset_C2,family=poisson,id=Subject..,corstr="unstructured") | | | | |
| --- | --- | --- | --- | --- |
| Coefficients | Estimate | Std.err | Wald | Pr(>\|W\|) |
| (Intercept) | 2.56 | 0.10 | 659.35 | <2e-16 *** |
| PlayOrder_1CR_2RC2 | -0.12 | 0.16 | 0.51 | 0.48 |
| Opponent2 | -.08 | 0.11 | 0.62 | 0.43 |
| Opponent3 | -0.30 | 0.12 | 6.47 | .011 * |
| PlayOrder_1CR_2RC2:Opponent2 | 0.22 | 0.15 | 2.24 | 0.14 |
| PlayOrder_1CR_2RC2:Opponent3 | -.04 | 0.17 | .04 | 0.84 |

Note. Significance level is indicated as follows: 0 (***), 0.001 (**), 0.01 (*), 0.05 (.), 0.1 ( ), 1

Table S2. Gee model on defection choices counts

| geeglm(Count~PlayOrder_1CR_2RC * Opponent,data=Subset_D2,family=poisson,id=Subject..,corstr="unstructured") | | | | |
| --- | --- | --- | --- | --- |
| Coefficients | Estimate | Std.err | Wald | Pr(>\|W\|) |
| (Intercept) | 2.82 | .08 | 1304.43 | <2e-16 *** |
| PlayOrder_1CR_2RC2 | .08 | 0.11 | 0.52 | 0.47 |
| Opponent2 | .06 | .07 | 0.65 | 0.42 |
| Opponent3 | 0.19 | .07 | 7.04 | .008 ** |
| PlayOrder_1CR_2RC2:Opponent2 | -0.16 | 0.11 | 2.09 | 0.15 |
| PlayOrder_1CR_2RC2:Opponent3 | -.02 | .08 | 0.05 | 0.83 |

Note. Significance level is indicated as follows: 0 (***), 0.001 (**), 0.01 (*), 0.05 (.), 0.1 ( ), 1

Table S3. Gee model on probability of cooperate after a CC outcome counts

| geeglm(CountProb~PlayOrder_1CR_2RC * Opponent+ offset(log(offsets)),data=Subset_CCpC5,family=poisson, id=Subject..,corstr="unstructured") | | | | |
| --- | --- | --- | --- | --- |
| Coefficients | Estimate | Std.err | Wald | Pr(>\|W\|) |
| (Intercept) | -0.19 | .05 | 14.98 | .00011 *** |
| PlayOrder_1CR_2RC2 | -.04 | 0.10 | 0.13 | 0.72 |
| Opponent2 | -.01 | .04 | .05 | 0.82 |
| Opponent3 | -.08 | .08 | 0.86 | 0.35 |
| PlayOrder_1CR_2RC2:Opponent2 | .04 | .07 | 0.32 | 0.57 |
| PlayOrder_1CR_2RC2:Opponent3 | .08 | 0.11 | 0.56 | 0.45 |

Note. Significance level is indicated as follows: 0 (***), 0.001 (**), 0.01 (*), 0.05 (.), 0.1 ( ), 1

Table S4. Gee model on probability of cooperate after a CD outcome counts

| geeglm(geeglm(CountProb~PlayOrder_1CR_2RC*Opponent + offset(log(offsets)),data=Subset_CDpC5,family=poisson,  id=Subject..,corstr="unstructured") | | | | |
| --- | --- | --- | --- | --- |
| Coefficients | Estimate | Std.err | Wald | Pr(>\|W\|) |
| (Intercept) | -0.64 | 0.15 | 18.80 | 1.4e-05 *** |
| PlayOrder_1CR_2RC2 | .035 | 0.27 | .02 | 0.90 |
| Opponent2 | 0.10 | 0.13 | 0.60 | 0.44 |
| Opponent3 | -.02 | 0.14 | .02 | 0.88 |
| PlayOrder_1CR_2RC2:Opponent2 | -.01 | 0.18 | .00 | 0.96 |
| PlayOrder_1CR_2RC2:Opponent3 | 0.67 | 0.33 | 4.13 | .04 * |

Note. Significance level is indicated as follows: 0 (***), 0.001 (**), 0.01 (*), 0.05 (.), 0.1 ( ), 1

Table S5. Gee model on probability of cooperate after a DC outcome counts

| geeglm(CountProb~PlayOrder_1CR_2RC * Opponent+ offset(log(offsets)),data=Subset_DCpC5,family=poisson,  id=Subject.., corstr="unstructured") | | | | |
| --- | --- | --- | --- | --- |
| Coefficients | Estimate | Std.err | Wald | Pr(>\|W\|) |
| (Intercept) | -0.39 | 0.11 | 11.59 | .0007 *** |
| PlayOrder_1CR_2RC2 | .06 | 0.14 | 0.20 | 0.66 |
| Opponent2 | -0.11 | 0.15 | 0.48 | 0.49 |
| Opponent3 | 0.19 | 0.14 | 2.01 | 0.16 |
| PlayOrder_1CR_2RC2:Opponent2 | .07 | 0.19 | 0.15 | 0.70 |
| PlayOrder_1CR_2RC2:Opponent3 | -0.49 | 0.26 | 3.51 | .06 |

Note. Significance level is indicated as follows: 0 (***), 0.001 (**), 0.01 (*), 0.05 (.), 0.1 ( ), 1

Table S6. Gee model on probability of cooperate after a DD outcome counts

| geeglm(CountProb~PlayOrder_1CR_2RC * Opponent+ offset(log(offsets)),data=Subset_DDpC5,family=poisson, id=Subject..,corstr="unstructured") | | | | |
| --- | --- | --- | --- | --- |
| Coefficients | Estimate | Std.err | Wald | Pr(>\|W\|) |
| (Intercept) | -1.51 | 0.15 | 101.11 | <2e-16 *** |
| PlayOrder_1CR_2RC2 | -0.15 | 0.22 | 0.45 | 0.50 |
| Opponent2 | -0.34 | 0.26 | 1.65 | 0.20 |
| Opponent3 | -0.28 | 0.25 | 1.33 | 0.25 |
| PlayOrder_1CR_2RC2:Opponent2 | 0.68 | 0.32 | 4.34 | .037 * |
| PlayOrder_1CR_2RC2:Opponent3 | .04 | 0.32 | .01 | 0.91 |

Note. Significance level is indicated as follows: 0 (***), 0.001 (**), 0.01 (*), 0.05 (.), 0.1 ( ), 1

**Additional Results**

**Defection choice frequency**

***Defection.*** Defection toward the human opponent was 16% lower than toward the roulette (*p* < 0.001) and defection toward computer was 18% lower than toward the roulette (*p* < 0.001). No defection differences were found between human and computer opponents (*p* = 0.99). No significant effect of the play-order was observed (*p* = 0.83). However, an interaction between opponents and play-order indicated how defection with human opponents was 17% lower than with the roulette (*p* = 0.02) in play-order HCR and 15% lower in play-order HRC (*p* < 0.001). In addition, only in play-order HRC, the defection toward the computer was 24% lower than toward the roulette (*p* = 0.003). See Table S7.

**Table S7.** Defection choice frequency estimated marginal means, and the effect of opponent on cooperation choice (pairwise comparisons) per each play-order. Asterisks signal statistically significant effects (Bonferroni-corrected p < .05), accompanied by their direction. SE = standard error; LCL/UCL = lower/upper confidence levels.

| **Defection choice frequency** | | | | | | | | | |
| --- | --- | --- | --- | --- | --- | --- | --- | --- | --- |
| **Opponent** | **Play-order** | **Rate** | | **SE** | | | **LCL** | **UCL** | |
| Human | HCR | 16.9 | | 1.32 | | | 14.5 | 19.6 | |
|  | HRC | 18.3 | | 1.50 | | | 15.6 | 21.5 | |
| Computer | HCR | 17.9 | | 1.49 | | | 15.2 | 21.1 | |
|  | HRC | 16.6 | | 1.93 | | | 13.2 | 20.8 | |
| Roulette | HCR | 20.3 | | 1.34 | | | 17.8 | 23.1 | |
|  | HRC | 21.6 | | 1.87 | | | 18.3 | 25.6 | |
| **Effect of opponent on defection choice frequency per play-order sequence** | | | | | | | | | |
| **Opponent Contrasts** | | **ratio** | **SE** | | **Z ratio** | **p value** | | | **Direction** |
| HCR | Human vs Computer | 0.94 | .07 | | 0.80 | .99 | | |  |
|  | Human vs Roulette | 0.83 | .06 | | -2.65 | .02* | | | (Hum < Rou) |
|  | Computer vs Roulette | 0.88 | .06 | | -1.90 | .17 | | |  |
| HRC | Human vs Computer | 1.11 | .09 | | 1.22 | .67 | | |  |
|  | Human vs Roulette | 0.85 | 03 | | -4.26 | <.0001* | | | (Hum < Rou) |
|  | Computer vs Roulette | 0.76 | .06 | | -3.33 | .003* | | | (Com < Rou) |

**Table S8.** Probability of cooperation after a cooperation-cooperation (CC) outcome estimates marginal means (EMMs), and effect of opponent on the probability of a cooperation after a CC outcome (pairwise comparisons) in each play order. SE = standard error; LCL/UCL = lower/upper confidence levels.

| **Transitional probability of a cooperation after CC** | | | | | | |
| --- | --- | --- | --- | --- | --- | --- |
| **Opponent** | **Play-order** | **Rate** | **SE** | | **LCL** | **UCL** |
| Human | HCR | 6.37 | 0.32 | | 5.77 | 7.03 |
|  | HRC | 6.14 | 0.53 | | 5.18 | 7.28 |
| Computer | HCR | 6.30 | 0.32 | | 5.70 | 6.97 |
|  | HRC | 6.33 | 0.33 | | 5.72 | 7.00 |
| Roulette | HCR | 5.89 | 0.56 | | 4.88 | 7.10 |
|  | HRC | 6.15 | 0.43 | | 5.37 | 7.04 |
| **Effect of opponent on transitional probability of cooperation after CC per play-order** | | | | | | |
| **Opponent Contrasts** | | **Ratio** | | **SE** | **Z ratio** | **p value** |
| HCR | Human vs Computer | 1.01 | | .04 | 0.23 | .99 |
|  | Human vs Roulette | 1.08 | | .09 | 0.93 | .99 |
|  | Computer vs Roulette | 1.07 | | .08 | 0.89 | .99 |
| HRC | Human vs Computer | 0.97 | | .05 | -0.54 | .99 |
|  | Human vs Roulette | 0.99 | | .06 | -.02 | .99 |
|  | Computer vs Roulette | 1.03 | | .04 | 0.77 | .99 |
| **Main effect of opponent on transitional probability of cooperation after CC** | | | | | | |
| **Opponent Contrasts** | | **Ratio** | | **SE** | **Z ratio** | **p value** |
| Human vs Computer | | 0.99 | | .03 | -0.28 | .99 |
| Human vs Roulette | | 1.04 | | .05 | 0.73 | .99 |
| Computer vs Roulette | | 1.05 | | .04 | 1.14 | .77 |

**Table S9.** Probability of cooperation after a cooperation-defection (CD) outcome estimates marginal means (EMMs), and effect of opponent on the probability of a cooperation after a CD outcome (pairwise comparisons), in each play order. SE = standard error; LCL/UCL = lower/upper confidence levels.

| **Transition probability of cooperation after CD** | | | | | | | |
| --- | --- | --- | --- | --- | --- | --- | --- |
| **Opponent** | **Play-order** | **Rate** | **SE** | | **LCL** | | **UCL** |
| Human | HCR | 2.99 | 0.44 | | 2.24 | | 4.00 |
|  | HRC | 3.10 | 0.69 | | 2.01 | | 4.79 |
| Computer | HCR | 3.31 | 0.43 | | 2.56 | | 4.26 |
|  | HRC | 3.39 | 0.51 | | 2.53 | | 4.55 |
| Roulette | HCR | 2.93 | 0.49 | | 2.11 | | 4.07 |
|  | HRC | 5.93 | 1.08 | | 4.16 | | 8.47 |
| **Effect of opponent on transitional probability of cooperation after CD per play-order** | | | | | | | |
| **Opponent Contrasts** | | **Ratio** | | **SE** | **Z ratio** | | **p value** |
| HCR | Human vs Computer | 0.91 | | 0.12 | -0.78 | | .99 |
|  | Human vs Roulette | 1.02 | | 0.14 | 0.16 | | .99 |
|  | Computer vs Roulette | 1.13 | | .09 | 1.58 | | .34 |
| HRC | Human vs Computer | 0.91 | | 0.11 | -0.73 | | .99 |
|  | Human vs Roulette | 0.52 | | 0.16 | -2.17 | | .09 |
|  | Computer vs Roulette | 0.57 | | 0.14 | -2.25 | | .07 |
| **Main effect of opponent on transitional probability of cooperation after CD** | | | | | | | |
| **Opponent Contrasts** | | **Ratio** | | **SE** | **Z ratio** | **p value** | |
| Human vs Computer | | 0.91 | | .08 | -1.07 | 0.86 | |
| Human vs Roulette | | 0.73 | | 0.12 | -1.91 | 0.17 | |
| Computer vs Roulette | | 0.80 | | 0.10 | -1.69 | .27 | |

**Table S10.** Probability of cooperation after a defection-cooperation (DC) outcome estimates marginal means (EMMs) and effect of opponent on the probability of a cooperation after a DC outcome (pairwise comparisons), in each play order. Asterisks signal statistically significant effects (Bonferroni-corrected p < 0.05), accompanied by their direction. SE = standard error; LCL/UCL = lower/upper confidence levels.

| **Transitional probability of cooperation after DC** | | | | | | | | | | | |
| --- | --- | --- | --- | --- | --- | --- | --- | --- | --- | --- | --- |
| **Opponent** | **Play-order** | **Rate** | | | | **SE** | **LCL** | | | | **UCL** |
| Human | HCR | 1.65 | | | | 0.19 | 1.32 | | | | 2.07 |
|  | HRC | 1.76 | | | | 0.14 | 1.50 | | | | 2.07 |
| Computer | HCR | 1.49 | | | | 0.21 | 1.13 | | | | 1.96 |
|  | HRC | 1.70 | | | | 0.16 | 1.42 | | | | 2.04 |
| Roulette | HCR | 2.01 | | | | 0.17 | 1.70 | | | | 2.37 |
|  | HRC | 1.31 | | | | 0.29 | 0.85 | | | | 2.02 |
| **Effect of opponent on transitional probability of cooperation after DC per play-order** | | | | | | | | | | | |
| **Opponent Contrasts** | | **Ratio** | | | **SE** | | **Z ratio** | | | **p value** | |
| HCR | Human vs Computer | 1.11 | | | 0.17 | | 0.69 | | | .99 | |
|  | Human vs Roulette | 0.82 | | | 0.11 | | -1.42 | | | .47 | |
|  | Computer vs Roulette | 0.74 | | | 0.10 | | -2.15 | | | .10 | |
| HRC | Human vs Computer | 1.04 | | | 0.11 | | 0.32 | | | .99 | |
|  | Human vs Roulette | 1.34 | | | 0.30 | | 1.32 | | | .56 | |
|  | Computer vs Roulette | 1.29 | | | 0.28 | | 1.18 | | | .71 | |
| **Main effect of opponent on transitional probability of cooperation after DC** | | | | | | | | | | | |
| **Opponent Contrasts** | | | **Ratio** | **SE** | | | | **Z ratio** | **p value** | | |
| Human vs Computer | | | 1.07 | 0.10 | | | | 0.75 | .99 | | |
| Human vs Roulette | | | 1.05 | 0.14 | | | | 0.38 | .99 | | |
| Computer vs Roulette | | | 0.98 | 0.13 | | | | -0.16 | .99 | | |

**Influence of subjective ratings on the choice frequencies per opponent.**

See attachment 1 and 2 for the result tables displaying the significant choice frequencies rate ratio in which the confidence interval did not contain 1 (i.e., index of no difference between the rate ratio of the pair of opponent analyzed in the contrast, at specific values of the subjective ratings). Below, the results are summarized indicating for which subjective rating values the choice frequencies rate ratio between the opponents is significantly different and in which direction.

**Agency**

**Agency: Cooperative choices**

- for values of 1.00 to 2.57, the (cooperative choice) rate ratio favors the human *vs* the roulette opponent (in a range from 35% to 92% higher),
- for values of 6.71 to 7.00 the (cooperative choice) rate ratio favors the roulette *vs* the human opponent (in a range from 49% to 46% higher).
- for values of 1.00 to 2.29 the (cooperative choice) rate ratio favors the computer vs the roulette opponent (in a range from 31% to 67% higher).

**Experience**

**Experience: Cooperative choices**

- for values of 1.09 to 1.90 the rate ratio favors the human vs the roulette opponent (in a range from 50% to 73% higher),
- for values of 1.00 to 1.45 the rate ratio favors the computer vs the roulette opponent (in a range from 39% to 53% higher).

**Experience: Defection choices**

- for values of 1.00 to 1.82 the rate ratio favors the roulette vs the computer opponent (in a range from 18% to 20% higher).

**Familiarity**

**Familiarity: Cooperative choices**

- for values of 5.00 to 9.00 the rate ratio favors the computer vs the human opponent (in a range from 20% to 44% higher).
- for values of 1.00 to 4.00 the rate ratio favors the human vs the roulette opponent (in a range from 17% to 66% higher).
- for values of 9.00 the rate ratio favors the roulette vs the human opponent (34% higher).
- for values of 1.00 to 5.00 the rate ratio favors the computer vs the roulette opponent (in a range from 31% to 45% higher).

**Familiarity: Defection choices**

- for values of 5.00 to 9.00 the rate ratio favors the human vs the computer opponent (in a range from 21% to 55% higher).
- for values of 1.00 to 5.00 the rate ratio favors the roulette vs the human opponent (in a range from 10% to 18% higher).
- for values of 1.00 to 9.00 the rate ratio favors the roulette vs the computer opponent (in a range from 35% to 13% higher).

**Human-Likeness**

**Human-Likeness: Cooperative choices**

- for values of 5.00 to 9.00 the rate ratio favors the computer vs the human opponent (in a range from 21% to 48% higher).
- for values of 1.00 to 4.00 the rate ratio favors the human vs the roulette opponent (in a range from 30% to 50% higher).
- for values of 2.00 to 9.00 the rate ratio favors the computer vs the roulette opponent (in a range from 32% to 98% higher).

**Human-Likeness : Defection choices**

- for values of 4.00 to 9.00 the rate ratio favors the human vs the computer opponent (in a range from 15% to 72% higher).
- for values of 1.00 to 5.00 the rate ratio favors the roulette vs the human opponent (in a range from 13% to 16% higher).
- for values of 2.00 to 9.00 the rate ratio favors the roulette vs the computer opponent (in a range from 13% to 47% higher).

**Influence of subjective ratings on the choice frequencies per opponent and play-order sequence**

**Agency**

**Agency: Cooperative choices**

- Playorder HRC, for values of 1.00 to 2.29 the rate ratio favors the computer vs the roulette opponent (in a range from 43% to 108% higher).

**Experience**

**Experience: Cooperative choices**

- Playorder HCR, for values of 1.82 to 3.27 the rate ratio favors the human vs the computer opponent (in a range from 62% to 193% higher).
- Playorder HCR, for values of 1.91 to 3.55 the rate ratio favors the human vs the roulette opponent (in a range from 47% to 55% higher).
- Playorder HCR, for values of 1 the rate ratio favors the computer vs the roulette opponent (38% higher).
- Playorder HRC, for values of 1 to 1.45 the rate ratio favors the computer vs the roulette opponent (70% higher).

**Experience: Defection choices**

- Playorder HCR, for values of 2.18 to 3.91 the rate ratio favors the computer vs the human opponent (in a range from 23% to 41% higher).
- Playorder HRC, for values of 1.18 to 1.91 the rate ratio favors the human vs the computer opponent (in a range from 34% to 39% higher).
- Playorder HCR, for values of 2.55 to 3.82 the rate ratio favors the roulette vs the human opponent (in a range from 20% to 22% higher).
- Playorder HRC, for values of 1 to 1.91 the rate ratio favors the roulette vs the computer opponent (in a range from 23% to 31% higher).

**Familiarity**

**Familiarity: Cooperative choices**

- Playorder HRC, for values of 4 to 9 the rate ratio favors the computer vs the human opponent (in a range from 21% to 48% higher).
- Playorder HCR, for values of 1 to 4 the rate ratio favors the human vs the roulette opponent (in a range from 18% to 43% higher).
- Playorder HRC, for values of 1 to 3 the rate ratio favors the human vs the roulette opponent (in a range from 38% to 93% higher), while for values of 8 to 9 the rate ratio favors the roulette vs the human opponent (in a range from 40% to 50% higher).
- Playorder HRC, for values of 1 to 4 the rate ratio favors the computer vs the roulette opponent (in a range from 55% to 68% higher).

**Familiarity: Defection choices**

- Playorder HRC, for values of 3 to 9 the rate ratio favors the human vs the computer opponent (in a range from 16% to 103% higher).
- Playorder HCR, for values of 1 to 5 the rate ratio favors the roulette vs the human opponent (in a range from 12% to 16% higher).
- Playorder HRC, for values of 1 to 4 the rate ratio favors the roulette vs the human opponent (in a range from 11% to 20% higher).
- Playorder HCR, for values of 3 to 4 the rate ratio favors the roulette vs the computer opponent (in a range from 12% to 14% higher).
- Playorder HRC, for values of 1 to 7 the rate ratio favors the roulette vs the computer opponent (in a range from 18% to 41% higher).

**Human-Likeness**

**Human-Likeness: Cooperative choices**

- Playorder HRC, for values of 4 to 9 the rate ratio favors the computer vs the human opponent (in a range from 22% to 54% higher).
- Playorder HCR, for values of 8 to 9 the rate ratio favors the computer vs the human opponent (in a range from 36% to 43% higher).
- Playorder HCR, for values of 1 to 4 the rate ratio favors the human vs the roulette opponent (in a range from 28% to 52% higher).
- Playorder HRC, for values of 2 to 3 the rate ratio favors the human vs the roulette opponent (in a range from 36% to 42% higher).
- Playorder HCR, for values of 3 to 9 the rate ratio favors the computer vs the roulette opponent (in a range from 25% to 68% higher).
- Playorder HRC, for values of 2 to 6 the rate ratio favors the computer vs the roulette opponent (in a range from 48% to 91% higher).

**Human-Likeness: Defection choices**

- Playorder HRC, for values of 4 to 9 the rate ratio favors the human vs the computer opponent (in a range from 23% to 95% higher).
- Playorder HRC, for values of 2 to 4 the rate ratio favors the roulette vs the human opponent (in a range from 15% to 16% higher).
- Playorder HCR, for values of 3 to 9 the rate ratio favors the roulette vs the computer opponent (in a range from 13% to 37% higher).
- Playorder HRC, for values of 2 to 9 the rate ratio favors the roulette vs the computer opponent (in a range from 18% to 56% higher).

**Attachment 1**

Significant choice frequencies rate ratio in which the confidence interval did not contain 1 (i.e., index of no difference between the rate ratio of the pair of opponent analyzed in the contrast, at specific values of the subjective ratings).

| **Questionnaire Type** | **Choice Count** | **Contrast** | | | **Questionnaire Rating** | **Ratio** | **LCL** | **UCL** |
| --- | --- | --- | --- | --- | --- | --- | --- | --- |
| Agency | Cooperation count | HUM | vs | ROU | 1.00 | 1.92 | 1.18 | 3.14 |
| Agency | Cooperation count | HUM | vs | ROU | 1.29 | 1.81 | 1.15 | 2.83 |
| Agency | Cooperation count | HUM | vs | ROU | 1.43 | 1.75 | 1.14 | 2.69 |
| Agency | Cooperation count | HUM | vs | ROU | 1.57 | 1.70 | 1.12 | 2.56 |
| Agency | Cooperation count | HUM | vs | ROU | 1.71 | 1.64 | 1.11 | 2.43 |
| Agency | Cooperation count | HUM | vs | ROU | 1.86 | 1.59 | 1.09 | 2.32 |
| Agency | Cooperation count | HUM | vs | ROU | 2.00 | 1.54 | 1.08 | 2.20 |
| Agency | Cooperation count | HUM | vs | ROU | 2.14 | 1.49 | 1.06 | 2.10 |
| Agency | Cooperation count | HUM | vs | ROU | 2.29 | 1.45 | 1.05 | 2.00 |
| Agency | Cooperation count | HUM | vs | ROU | 2.57 | 1.36 | 1.01 | 1.83 |
| Agency | Cooperation count | HUM | vs | ROU | 6.71 | 0.54 | 0.30 | 0.98 |
| Agency | Cooperation count | HUM | vs | ROU | 6.86 | 0.53 | 0.28 | 0.97 |
| Agency | Cooperation count | HUM | vs | ROU | 7.00 | 0.51 | 0.27 | 0.96 |
| Agency | Cooperation count | COM | vs | ROU | 1.00 | 1.67 | 1.08 | 2.59 |
| Agency | Cooperation count | COM | vs | ROU | 1.29 | 1.58 | 1.07 | 2.34 |
| Agency | Cooperation count | COM | vs | ROU | 1.43 | 1.54 | 1.06 | 2.23 |
| Agency | Cooperation count | COM | vs | ROU | 1.57 | 1.50 | 1.06 | 2.12 |
| Agency | Cooperation count | COM | vs | ROU | 1.71 | 1.46 | 1.05 | 2.02 |
| Agency | Cooperation count | COM | vs | ROU | 1.86 | 1.42 | 1.04 | 1.93 |
| Agency | Cooperation count | COM | vs | ROU | 2.00 | 1.38 | 1.03 | 1.84 |
| Agency | Cooperation count | COM | vs | ROU | 2.14 | 1.34 | 1.02 | 1.76 |
| Agency | Cooperation count | COM | vs | ROU | 2.29 | 1.31 | 1.01 | 1.69 |
| Experience | Cooperation count | HUM | vs | ROU | 1.09 | 1.50 | 1.01 | 2.23 |
| Experience | Cooperation count | HUM | vs | ROU | 1.18 | 1.52 | 1.04 | 2.24 |
| Experience | Cooperation count | HUM | vs | ROU | 1.27 | 1.55 | 1.06 | 2.26 |
| Experience | Cooperation count | HUM | vs | ROU | 1.36 | 1.57 | 1.07 | 2.30 |
| Experience | Cooperation count | HUM | vs | ROU | 1.45 | 1.60 | 1.08 | 2.36 |
| Experience | Cooperation count | HUM | vs | ROU | 1.55 | 1.62 | 1.08 | 2.44 |
| Experience | Cooperation count | HUM | vs | ROU | 1.64 | 1.65 | 1.07 | 2.53 |
| Experience | Cooperation count | HUM | vs | ROU | 1.73 | 1.67 | 1.06 | 2.64 |
| Experience | Cooperation count | HUM | vs | ROU | 1.82 | 1.70 | 1.05 | 2.76 |
| Experience | Cooperation count | HUM | vs | ROU | 1.91 | 1.73 | 1.03 | 2.90 |
| Experience | Cooperation count | COM | vs | ROU | 1.00 | 1.53 | 1.19 | 1.97 |
| Experience | Cooperation count | COM | vs | ROU | 1.09 | 1.50 | 1.18 | 1.90 |
| Experience | Cooperation count | COM | vs | ROU | 1.18 | 1.47 | 1.17 | 1.86 |
| Experience | Cooperation count | COM | vs | ROU | 1.27 | 1.44 | 1.14 | 1.83 |
| Experience | Cooperation count | COM | vs | ROU | 1.36 | 1.41 | 1.10 | 1.82 |
| Experience | Cooperation count | COM | vs | ROU | 1.45 | 1.39 | 1.05 | 1.83 |
| Experience | Defection count | COM | vs | ROU | 1.00 | 0.80 | 0.69 | 0.94 |
| Experience | Defection count | COM | vs | ROU | 1.09 | 0.81 | 0.70 | 0.93 |
| Experience | Defection count | COM | vs | ROU | 1.18 | 0.81 | 0.71 | 0.92 |
| Experience | Defection count | COM | vs | ROU | 1.27 | 0.81 | 0.72 | 0.91 |
| Experience | Defection count | COM | vs | ROU | 1.36 | 0.81 | 0.72 | 0.91 |
| Experience | Defection count | COM | vs | ROU | 1.45 | 0.81 | 0.72 | 0.92 |
| Experience | Defection count | COM | vs | ROU | 1.55 | 0.81 | 0.71 | 0.93 |
| Experience | Defection count | COM | vs | ROU | 1.64 | 0.81 | 0.71 | 0.94 |
| Experience | Defection count | COM | vs | ROU | 1.73 | 0.82 | 0.69 | 0.96 |
| Experience | Defection count | COM | vs | ROU | 1.82 | 0.82 | 0.68 | 0.98 |
| Human-likeness | Cooperation count | HUM | vs | COM | 5.00 | 0.79 | 0.68 | 0.91 |
| Human-likeness | Cooperation count | HUM | vs | COM | 6.00 | 0.71 | 0.58 | 0.85 |
| Human-likeness | Cooperation count | HUM | vs | COM | 7.00 | 0.64 | 0.49 | 0.82 |
| Human-likeness | Cooperation count | HUM | vs | COM | 8.00 | 0.57 | 0.41 | 0.80 |
| Human-likeness | Cooperation count | HUM | vs | COM | 9.00 | 0.52 | 0.34 | 0.78 |
| Human-likeness | Cooperation count | HUM | vs | ROU | 1.00 | 1.50 | 1.11 | 2.02 |
| Human-likeness | Cooperation count | HUM | vs | ROU | 2.00 | 1.43 | 1.13 | 1.80 |
| Human-likeness | Cooperation count | HUM | vs | ROU | 3.00 | 1.36 | 1.13 | 1.64 |
| Human-likeness | Cooperation count | HUM | vs | ROU | 4.00 | 1.30 | 1.07 | 1.56 |
| Human-likeness | Cooperation count | COM | vs | ROU | 2.00 | 1.32 | 1.09 | 1.61 |
| Human-likeness | Cooperation count | COM | vs | ROU | 3.00 | 1.40 | 1.18 | 1.67 |
| Human-likeness | Cooperation count | COM | vs | ROU | 4.00 | 1.48 | 1.22 | 1.81 |
| Human-likeness | Cooperation count | COM | vs | ROU | 5.00 | 1.57 | 1.22 | 2.02 |
| Human-likeness | Cooperation count | COM | vs | ROU | 6.00 | 1.66 | 1.21 | 2.29 |
| Human-likeness | Cooperation count | COM | vs | ROU | 7.00 | 1.76 | 1.19 | 2.62 |
| Human-likeness | Cooperation count | COM | vs | ROU | 8.00 | 1.87 | 1.16 | 3.01 |
| Human-likeness | Cooperation count | COM | vs | ROU | 9.00 | 1.98 | 1.13 | 3.46 |
| Human-likeness | Defection count | HUM | vs | COM | 4.00 | 1.15 | 1.02 | 1.29 |
| Human-likeness | Defection count | HUM | vs | COM | 5.00 | 1.24 | 1.09 | 1.43 |
| Human-likeness | Defection count | HUM | vs | COM | 6.00 | 1.35 | 1.13 | 1.61 |
| Human-likeness | Defection count | HUM | vs | COM | 7.00 | 1.46 | 1.17 | 1.83 |
| Human-likeness | Defection count | HUM | vs | COM | 8.00 | 1.59 | 1.21 | 2.08 |
| Human-likeness | Defection count | HUM | vs | COM | 9.00 | 1.72 | 1.24 | 2.38 |
| Human-likeness | Defection count | HUM | vs | ROU | 1.00 | 0.84 | 0.71 | 0.99 |
| Human-likeness | Defection count | HUM | vs | ROU | 2.00 | 0.85 | 0.74 | 0.97 |
| Human-likeness | Defection count | HUM | vs | ROU | 3.00 | 0.86 | 0.76 | 0.96 |
| Human-likeness | Defection count | HUM | vs | ROU | 4.00 | 0.86 | 0.77 | 0.96 |
| Human-likeness | Defection count | HUM | vs | ROU | 5.00 | 0.87 | 0.78 | 0.98 |
| Human-likeness | Defection count | COM | vs | ROU | 2.00 | 0.87 | 0.79 | 0.95 |
| Human-likeness | Defection count | COM | vs | ROU | 3.00 | 0.81 | 0.74 | 0.88 |
| Human-likeness | Defection count | COM | vs | ROU | 4.00 | 0.75 | 0.68 | 0.84 |
| Human-likeness | Defection count | COM | vs | ROU | 5.00 | 0.70 | 0.61 | 0.81 |
| Human-likeness | Defection count | COM | vs | ROU | 6.00 | 0.65 | 0.54 | 0.79 |
| Human-likeness | Defection count | COM | vs | ROU | 7.00 | 0.61 | 0.48 | 0.77 |
| Human-likeness | Defection count | COM | vs | ROU | 8.00 | 0.57 | 0.43 | 0.75 |
| Human-likeness | Defection count | COM | vs | ROU | 9.00 | 0.53 | 0.38 | 0.74 |
| Familiarity | Cooperation count | HUM | vs | COM | 5.00 | 0.80 | 0.66 | 0.98 |
| Familiarity | Cooperation count | HUM | vs | COM | 6.00 | 0.73 | 0.57 | 0.95 |
| Familiarity | Cooperation count | HUM | vs | COM | 7.00 | 0.67 | 0.48 | 0.92 |
| Familiarity | Cooperation count | HUM | vs | COM | 8.00 | 0.61 | 0.41 | 0.90 |
| Familiarity | Cooperation count | HUM | vs | COM | 9.00 | 0.56 | 0.35 | 0.88 |
| Familiarity | Cooperation count | HUM | vs | ROU | 1.00 | 1.66 | 1.40 | 1.98 |
| Familiarity | Cooperation count | HUM | vs | ROU | 2.00 | 1.48 | 1.29 | 1.70 |
| Familiarity | Cooperation count | HUM | vs | ROU | 3.00 | 1.32 | 1.17 | 1.49 |
| Familiarity | Cooperation count | HUM | vs | ROU | 4.00 | 1.17 | 1.03 | 1.33 |
| Familiarity | Cooperation count | HUM | vs | ROU | 9.00 | 0.66 | 0.46 | 0.94 |
| Familiarity | Cooperation count | COM | vs | ROU | 1.00 | 1.45 | 1.16 | 1.82 |
| Familiarity | Cooperation count | COM | vs | ROU | 2.00 | 1.41 | 1.18 | 1.69 |
| Familiarity | Cooperation count | COM | vs | ROU | 3.00 | 1.38 | 1.17 | 1.62 |
| Familiarity | Cooperation count | COM | vs | ROU | 4.00 | 1.34 | 1.10 | 1.63 |
| Familiarity | Cooperation count | COM | vs | ROU | 5.00 | 1.31 | 1.02 | 1.68 |
| Familiarity | Defection count | HUM | vs | COM | 5.00 | 1.21 | 1.01 | 1.44 |
| Familiarity | Defection count | HUM | vs | COM | 6.00 | 1.28 | 1.03 | 1.60 |
| Familiarity | Defection count | HUM | vs | COM | 7.00 | 1.37 | 1.04 | 1.79 |
| Familiarity | Defection count | HUM | vs | COM | 8.00 | 1.45 | 1.06 | 2.00 |
| Familiarity | Defection count | HUM | vs | COM | 9.00 | 1.55 | 1.07 | 2.24 |
| Familiarity | Defection count | HUM | vs | ROU | 1.00 | 0.82 | 0.74 | 0.91 |
| Familiarity | Defection count | HUM | vs | ROU | 2.00 | 0.84 | 0.78 | 0.90 |
| Familiarity | Defection count | HUM | vs | ROU | 3.00 | 0.86 | 0.81 | 0.91 |
| Familiarity | Defection count | HUM | vs | ROU | 4.00 | 0.88 | 0.82 | 0.95 |
| Familiarity | Defection count | HUM | vs | ROU | 5.00 | 0.90 | 0.82 | 1.00 |
| Familiarity | Defection count | COM | vs | ROU | 1.00 | 0.87 | 0.77 | 0.98 |
| Familiarity | Defection count | COM | vs | ROU | 2.00 | 0.84 | 0.77 | 0.91 |
| Familiarity | Defection count | COM | vs | ROU | 3.00 | 0.81 | 0.73 | 0.89 |
| Familiarity | Defection count | COM | vs | ROU | 4.00 | 0.78 | 0.68 | 0.89 |
| Familiarity | Defection count | COM | vs | ROU | 5.00 | 0.75 | 0.62 | 0.91 |
| Familiarity | Defection count | COM | vs | ROU | 6.00 | 0.72 | 0.57 | 0.92 |
| Familiarity | Defection count | COM | vs | ROU | 7.00 | 0.70 | 0.51 | 0.94 |
| Familiarity | Defection count | COM | vs | ROU | 8.00 | 0.67 | 0.47 | 0.96 |
| Familiarity | Defection count | COM | vs | ROU | 9.00 | 0.65 | 0.42 | 0.99 |

**Attachment 2**

Significant choice frequencies rate ratio, by play-order, in which the confidence interval did not contain 1 (i.e., index of no difference between the rate ratio of the pair of opponent analyzed in the contrast, at specific values of the subjective ratings).

| **Questionnaire Type** | **Choice Count** | **Playorder** | **Contrast** | | | **Questionnaire Rating** | **Ratio** | **LCL** | **UCL** |
| --- | --- | --- | --- | --- | --- | --- | --- | --- | --- |
| Agency | Cooperation count | HRC | COM | vs | ROU | 1 | 2.08 | 1.28 | 3.39 |
| Agency | Cooperation count | HRC | COM | vs | ROU | 1.29 | 1.92 | 1.24 | 2.96 |
| Agency | Cooperation count | HRC | COM | vs | ROU | 1.43 | 1.84 | 1.21 | 2.78 |
| Agency | Cooperation count | HRC | COM | vs | ROU | 1.57 | 1.76 | 1.19 | 2.61 |
| Agency | Cooperation count | HRC | COM | vs | ROU | 1.71 | 1.69 | 1.16 | 2.46 |
| Agency | Cooperation count | HRC | COM | vs | ROU | 1.86 | 1.62 | 1.13 | 2.32 |
| Agency | Cooperation count | HRC | COM | vs | ROU | 2 | 1.56 | 1.1 | 2.2 |
| Agency | Cooperation count | HRC | COM | vs | ROU | 2.14 | 1.49 | 1.07 | 2.09 |
| Agency | Cooperation count | HRC | COM | vs | ROU | 2.29 | 1.43 | 1.03 | 1.99 |
| Familiarity | Cooperation count | HRC | HUM | vs | COM | 4 | 0.75 | 0.59 | 0.96 |
| Familiarity | Cooperation count | HRC | HUM | vs | COM | 5 | 0.65 | 0.48 | 0.9 |
| Familiarity | Cooperation count | HRC | HUM | vs | COM | 6 | 0.57 | 0.38 | 0.85 |
| Familiarity | Cooperation count | HRC | HUM | vs | COM | 7 | 0.49 | 0.3 | 0.81 |
| Familiarity | Cooperation count | HRC | HUM | vs | COM | 8 | 0.43 | 0.24 | 0.78 |
| Familiarity | Cooperation count | HRC | HUM | vs | COM | 9 | 0.37 | 0.18 | 0.74 |
| Familiarity | Cooperation count | HCR | HUM | vs | ROU | 1 | 1.43 | 1.09 | 1.87 |
| Familiarity | Cooperation count | HCR | HUM | vs | ROU | 2 | 1.34 | 1.09 | 1.66 |
| Familiarity | Cooperation count | HCR | HUM | vs | ROU | 3 | 1.26 | 1.06 | 1.49 |
| Familiarity | Cooperation count | HCR | HUM | vs | ROU | 4 | 1.18 | 1 | 1.39 |
| Familiarity | Cooperation count | HRC | HUM | vs | ROU | 1 | 1.93 | 1.55 | 2.42 |
| Familiarity | Cooperation count | HRC | HUM | vs | ROU | 2 | 1.64 | 1.37 | 1.95 |
| Familiarity | Cooperation count | HRC | HUM | vs | ROU | 3 | 1.38 | 1.17 | 1.63 |
| Familiarity | Cooperation count | HRC | HUM | vs | ROU | 8 | 0.6 | 0.37 | 0.95 |
| Familiarity | Cooperation count | HRC | HUM | vs | ROU | 9 | 0.5 | 0.29 | 0.87 |
| Familiarity | Cooperation count | HRC | COM | vs | ROU | 1 | 1.68 | 1.23 | 2.29 |
| Familiarity | Cooperation count | HRC | COM | vs | ROU | 2 | 1.63 | 1.25 | 2.13 |
| Familiarity | Cooperation count | HRC | COM | vs | ROU | 3 | 1.59 | 1.2 | 2.1 |
| Familiarity | Cooperation count | HRC | COM | vs | ROU | 4 | 1.55 | 1.1 | 2.18 |
| Familiarity | Defection count | HRC | HUM | vs | COM | 3 | 1.16 | 1.01 | 1.35 |
| Familiarity | Defection count | HRC | HUM | vs | COM | 4 | 1.28 | 1.05 | 1.56 |
| Familiarity | Defection count | HRC | HUM | vs | COM | 5 | 1.4 | 1.08 | 1.83 |
| Familiarity | Defection count | HRC | HUM | vs | COM | 6 | 1.54 | 1.1 | 2.15 |
| Familiarity | Defection count | HRC | HUM | vs | COM | 7 | 1.69 | 1.12 | 2.54 |
| Familiarity | Defection count | HRC | HUM | vs | COM | 8 | 1.85 | 1.14 | 3.01 |
| Familiarity | Defection count | HRC | HUM | vs | COM | 9 | 2.03 | 1.16 | 3.56 |
| Familiarity | Defection count | HCR | HUM | vs | ROU | 1 | 0.84 | 0.7 | 1 |
| Familiarity | Defection count | HCR | HUM | vs | ROU | 2 | 0.85 | 0.74 | 0.97 |
| Familiarity | Defection count | HCR | HUM | vs | ROU | 3 | 0.86 | 0.77 | 0.96 |
| Familiarity | Defection count | HCR | HUM | vs | ROU | 4 | 0.87 | 0.78 | 0.97 |
| Familiarity | Defection count | HCR | HUM | vs | ROU | 5 | 0.88 | 0.78 | 1 |
| Familiarity | Defection count | HRC | HUM | vs | ROU | 1 | 0.8 | 0.72 | 0.89 |
| Familiarity | Defection count | HRC | HUM | vs | ROU | 2 | 0.83 | 0.78 | 0.88 |
| Familiarity | Defection count | HRC | HUM | vs | ROU | 3 | 0.86 | 0.81 | 0.91 |
| Familiarity | Defection count | HRC | HUM | vs | ROU | 4 | 0.89 | 0.81 | 0.99 |
| Familiarity | Defection count | HCR | COM | vs | ROU | 3 | 0.88 | 0.79 | 0.98 |
| Familiarity | Defection count | HCR | COM | vs | ROU | 4 | 0.86 | 0.75 | 0.99 |
| Familiarity | Defection count | HRC | COM | vs | ROU | 1 | 0.82 | 0.69 | 0.98 |
| Familiarity | Defection count | HRC | COM | vs | ROU | 2 | 0.78 | 0.68 | 0.89 |
| Familiarity | Defection count | HRC | COM | vs | ROU | 3 | 0.74 | 0.63 | 0.87 |
| Familiarity | Defection count | HRC | COM | vs | ROU | 4 | 0.7 | 0.55 | 0.89 |
| Familiarity | Defection count | HRC | COM | vs | ROU | 5 | 0.66 | 0.48 | 0.92 |
| Familiarity | Defection count | HRC | COM | vs | ROU | 6 | 0.63 | 0.41 | 0.95 |
| Familiarity | Defection count | HRC | COM | vs | ROU | 7 | 0.59 | 0.35 | 0.99 |
| Human-likeness | Cooperation count | HCR | HUM | vs | COM | 8 | 0.64 | 0.42 | 0.98 |
| Human-likeness | Cooperation count | HCR | HUM | vs | COM | 9 | 0.57 | 0.34 | 0.96 |
| Human-likeness | Cooperation count | HRC | HUM | vs | COM | 4 | 0.78 | 0.65 | 0.93 |
| Human-likeness | Cooperation count | HRC | HUM | vs | COM | 5 | 0.7 | 0.59 | 0.83 |
| Human-likeness | Cooperation count | HRC | HUM | vs | COM | 6 | 0.63 | 0.49 | 0.82 |
| Human-likeness | Cooperation count | HRC | HUM | vs | COM | 7 | 0.57 | 0.39 | 0.83 |
| Human-likeness | Cooperation count | HRC | HUM | vs | COM | 8 | 0.52 | 0.31 | 0.86 |
| Human-likeness | Cooperation count | HRC | HUM | vs | COM | 9 | 0.46 | 0.24 | 0.89 |
| Human-likeness | Cooperation count | HCR | HUM | vs | ROU | 1 | 1.52 | 1.03 | 2.25 |
| Human-likeness | Cooperation count | HCR | HUM | vs | ROU | 2 | 1.44 | 1.04 | 1.98 |
| Human-likeness | Cooperation count | HCR | HUM | vs | ROU | 3 | 1.36 | 1.05 | 1.76 |
| Human-likeness | Cooperation count | HCR | HUM | vs | ROU | 4 | 1.28 | 1.03 | 1.6 |
| Human-likeness | Cooperation count | HRC | HUM | vs | ROU | 2 | 1.42 | 1.02 | 1.97 |
| Human-likeness | Cooperation count | HRC | HUM | vs | ROU | 3 | 1.36 | 1.04 | 1.78 |
| Human-likeness | Cooperation count | HCR | COM | vs | ROU | 3 | 1.25 | 1.02 | 1.53 |
| Human-likeness | Cooperation count | HCR | COM | vs | ROU | 4 | 1.31 | 1.1 | 1.56 |
| Human-likeness | Cooperation count | HCR | COM | vs | ROU | 5 | 1.38 | 1.14 | 1.67 |
| Human-likeness | Cooperation count | HCR | COM | vs | ROU | 6 | 1.45 | 1.13 | 1.86 |
| Human-likeness | Cooperation count | HCR | COM | vs | ROU | 7 | 1.52 | 1.11 | 2.1 |
| Human-likeness | Cooperation count | HCR | COM | vs | ROU | 8 | 1.6 | 1.07 | 2.39 |
| Human-likeness | Cooperation count | HCR | COM | vs | ROU | 9 | 1.68 | 1.04 | 2.74 |
| Human-likeness | Cooperation count | HRC | COM | vs | ROU | 2 | 1.48 | 1.1 | 1.98 |
| Human-likeness | Cooperation count | HRC | COM | vs | ROU | 3 | 1.57 | 1.18 | 2.1 |
| Human-likeness | Cooperation count | HRC | COM | vs | ROU | 4 | 1.68 | 1.18 | 2.39 |
| Human-likeness | Cooperation count | HRC | COM | vs | ROU | 5 | 1.79 | 1.13 | 2.84 |
| Human-likeness | Cooperation count | HRC | COM | vs | ROU | 6 | 1.91 | 1.06 | 3.44 |
| Human-likeness | Defection count | HRC | HUM | vs | COM | 4 | 1.23 | 1.12 | 1.34 |
| Human-likeness | Defection count | HRC | HUM | vs | COM | 5 | 1.35 | 1.21 | 1.5 |
| Human-likeness | Defection count | HRC | HUM | vs | COM | 6 | 1.48 | 1.24 | 1.76 |
| Human-likeness | Defection count | HRC | HUM | vs | COM | 7 | 1.62 | 1.25 | 2.1 |
| Human-likeness | Defection count | HRC | HUM | vs | COM | 8 | 1.78 | 1.26 | 2.5 |
| Human-likeness | Defection count | HRC | HUM | vs | COM | 9 | 1.95 | 1.27 | 2.99 |
| Human-likeness | Defection count | HRC | HUM | vs | ROU | 2 | 0.84 | 0.72 | 0.99 |
| Human-likeness | Defection count | HRC | HUM | vs | ROU | 3 | 0.84 | 0.73 | 0.98 |
| Human-likeness | Defection count | HRC | HUM | vs | ROU | 4 | 0.85 | 0.72 | 0.99 |
| Human-likeness | Defection count | HCR | COM | vs | ROU | 3 | 0.87 | 0.77 | 0.97 |
| Human-likeness | Defection count | HCR | COM | vs | ROU | 4 | 0.82 | 0.71 | 0.95 |
| Human-likeness | Defection count | HCR | COM | vs | ROU | 5 | 0.78 | 0.64 | 0.95 |
| Human-likeness | Defection count | HCR | COM | vs | ROU | 6 | 0.74 | 0.58 | 0.95 |
| Human-likeness | Defection count | HCR | COM | vs | ROU | 7 | 0.7 | 0.52 | 0.95 |
| Human-likeness | Defection count | HCR | COM | vs | ROU | 8 | 0.67 | 0.46 | 0.96 |
| Human-likeness | Defection count | HCR | COM | vs | ROU | 9 | 0.63 | 0.41 | 0.97 |
| Human-likeness | Defection count | HRC | COM | vs | ROU | 2 | 0.82 | 0.71 | 0.96 |
| Human-likeness | Defection count | HRC | COM | vs | ROU | 3 | 0.75 | 0.67 | 0.85 |
| Human-likeness | Defection count | HRC | COM | vs | ROU | 4 | 0.69 | 0.59 | 0.8 |
| Human-likeness | Defection count | HRC | COM | vs | ROU | 5 | 0.63 | 0.51 | 0.77 |
| Human-likeness | Defection count | HRC | COM | vs | ROU | 6 | 0.58 | 0.44 | 0.76 |
| Human-likeness | Defection count | HRC | COM | vs | ROU | 7 | 0.53 | 0.37 | 0.75 |
| Human-likeness | Defection count | HRC | COM | vs | ROU | 8 | 0.48 | 0.31 | 0.74 |
| Human-likeness | Defection count | HRC | COM | vs | ROU | 9 | 0.44 | 0.26 | 0.73 |
| Experience | Cooperation count | HCR | HUM | vs | COM | 1.82 | 1.62 | 1.02 | 2.56 |
| Experience | Cooperation count | HCR | HUM | vs | COM | 1.91 | 1.68 | 1.04 | 2.71 |
| Experience | Cooperation count | HCR | HUM | vs | COM | 2.18 | 1.88 | 1.07 | 3.28 |
| Experience | Cooperation count | HCR | HUM | vs | COM | 2.55 | 2.18 | 1.07 | 4.42 |
| Experience | Cooperation count | HCR | HUM | vs | COM | 2.82 | 2.44 | 1.05 | 5.63 |
| Experience | Cooperation count | HCR | HUM | vs | COM | 2.91 | 2.53 | 1.05 | 6.11 |
| Experience | Cooperation count | HCR | HUM | vs | COM | 3 | 2.62 | 1.04 | 6.65 |
| Experience | Cooperation count | HCR | HUM | vs | COM | 3.09 | 2.72 | 1.03 | 7.23 |
| Experience | Cooperation count | HCR | HUM | vs | COM | 3.18 | 2.83 | 1.02 | 7.87 |
| Experience | Cooperation count | HCR | HUM | vs | COM | 3.27 | 2.93 | 1 | 8.58 |
| Experience | Cooperation count | HCR | HUM | vs | ROU | 1.91 | 1.55 | 1.01 | 2.37 |
| Experience | Cooperation count | HCR | HUM | vs | ROU | 2.18 | 1.54 | 1.08 | 2.19 |
| Experience | Cooperation count | HCR | HUM | vs | ROU | 2.55 | 1.52 | 1.14 | 2.03 |
| Experience | Cooperation count | HCR | HUM | vs | ROU | 2.82 | 1.51 | 1.15 | 1.97 |
| Experience | Cooperation count | HCR | HUM | vs | ROU | 2.91 | 1.5 | 1.14 | 1.97 |
| Experience | Cooperation count | HCR | HUM | vs | ROU | 3 | 1.5 | 1.14 | 1.97 |
| Experience | Cooperation count | HCR | HUM | vs | ROU | 3.09 | 1.49 | 1.12 | 1.98 |
| Experience | Cooperation count | HCR | HUM | vs | ROU | 3.18 | 1.49 | 1.11 | 2 |
| Experience | Cooperation count | HCR | HUM | vs | ROU | 3.27 | 1.48 | 1.09 | 2.02 |
| Experience | Cooperation count | HCR | HUM | vs | ROU | 3.36 | 1.48 | 1.07 | 2.05 |
| Experience | Cooperation count | HCR | HUM | vs | ROU | 3.45 | 1.48 | 1.04 | 2.08 |
| Experience | Cooperation count | HCR | HUM | vs | ROU | 3.55 | 1.47 | 1.02 | 2.12 |
| Experience | Cooperation count | HCR | COM | vs | ROU | 1 | 1.38 | 1.02 | 1.87 |
| Experience | Cooperation count | HRC | COM | vs | ROU | 1 | 1.7 | 1.14 | 2.55 |
| Experience | Cooperation count | HRC | COM | vs | ROU | 1.09 | 1.7 | 1.17 | 2.48 |
| Experience | Cooperation count | HRC | COM | vs | ROU | 1.18 | 1.7 | 1.18 | 2.46 |
| Experience | Cooperation count | HRC | COM | vs | ROU | 1.27 | 1.7 | 1.16 | 2.5 |
| Experience | Cooperation count | HRC | COM | vs | ROU | 1.36 | 1.7 | 1.12 | 2.59 |
| Experience | Cooperation count | HRC | COM | vs | ROU | 1.45 | 1.7 | 1.06 | 2.72 |
| Experience | Defection count | HCR | HUM | vs | COM | 2.18 | 0.77 | 0.6 | 0.99 |
| Experience | Defection count | HCR | HUM | vs | COM | 2.55 | 0.73 | 0.56 | 0.94 |
| Experience | Defection count | HCR | HUM | vs | COM | 2.82 | 0.7 | 0.52 | 0.93 |
| Experience | Defection count | HCR | HUM | vs | COM | 2.91 | 0.69 | 0.51 | 0.93 |
| Experience | Defection count | HCR | HUM | vs | COM | 3 | 0.68 | 0.5 | 0.93 |
| Experience | Defection count | HCR | HUM | vs | COM | 3.09 | 0.67 | 0.48 | 0.93 |
| Experience | Defection count | HCR | HUM | vs | COM | 3.18 | 0.66 | 0.47 | 0.94 |
| Experience | Defection count | HCR | HUM | vs | COM | 3.27 | 0.65 | 0.45 | 0.94 |
| Experience | Defection count | HCR | HUM | vs | COM | 3.36 | 0.64 | 0.44 | 0.95 |
| Experience | Defection count | HCR | HUM | vs | COM | 3.45 | 0.63 | 0.42 | 0.95 |
| Experience | Defection count | HCR | HUM | vs | COM | 3.55 | 0.63 | 0.41 | 0.96 |
| Experience | Defection count | HCR | HUM | vs | COM | 3.64 | 0.62 | 0.39 | 0.97 |
| Experience | Defection count | HCR | HUM | vs | COM | 3.82 | 0.6 | 0.37 | 0.98 |
| Experience | Defection count | HCR | HUM | vs | COM | 3.91 | 0.59 | 0.35 | 0.99 |
| Experience | Defection count | HRC | HUM | vs | COM | 1.18 | 1.39 | 1 | 1.94 |
| Experience | Defection count | HRC | HUM | vs | COM | 1.27 | 1.39 | 1.02 | 1.88 |
| Experience | Defection count | HRC | HUM | vs | COM | 1.36 | 1.38 | 1.04 | 1.83 |
| Experience | Defection count | HRC | HUM | vs | COM | 1.45 | 1.37 | 1.05 | 1.8 |
| Experience | Defection count | HRC | HUM | vs | COM | 1.55 | 1.37 | 1.06 | 1.77 |
| Experience | Defection count | HRC | HUM | vs | COM | 1.64 | 1.36 | 1.05 | 1.75 |
| Experience | Defection count | HRC | HUM | vs | COM | 1.73 | 1.35 | 1.05 | 1.75 |
| Experience | Defection count | HRC | HUM | vs | COM | 1.82 | 1.35 | 1.03 | 1.76 |
| Experience | Defection count | HRC | HUM | vs | COM | 1.91 | 1.34 | 1.01 | 1.78 |
| Experience | Defection count | HCR | HUM | vs | ROU | 2.55 | 0.78 | 0.63 | 0.97 |
| Experience | Defection count | HCR | HUM | vs | ROU | 2.82 | 0.79 | 0.66 | 0.94 |
| Experience | Defection count | HCR | HUM | vs | ROU | 2.91 | 0.79 | 0.66 | 0.94 |
| Experience | Defection count | HCR | HUM | vs | ROU | 3 | 0.79 | 0.67 | 0.93 |
| Experience | Defection count | HCR | HUM | vs | ROU | 3.09 | 0.79 | 0.67 | 0.93 |
| Experience | Defection count | HCR | HUM | vs | ROU | 3.18 | 0.79 | 0.67 | 0.93 |
| Experience | Defection count | HCR | HUM | vs | ROU | 3.27 | 0.79 | 0.67 | 0.94 |
| Experience | Defection count | HCR | HUM | vs | ROU | 3.36 | 0.79 | 0.67 | 0.94 |
| Experience | Defection count | HCR | HUM | vs | ROU | 3.45 | 0.79 | 0.67 | 0.95 |
| Experience | Defection count | HCR | HUM | vs | ROU | 3.55 | 0.8 | 0.66 | 0.96 |
| Experience | Defection count | HCR | HUM | vs | ROU | 3.64 | 0.8 | 0.66 | 0.97 |
| Experience | Defection count | HCR | HUM | vs | ROU | 3.82 | 0.8 | 0.64 | 0.99 |
| Experience | Defection count | HRC | COM | vs | ROU | 1 | 0.77 | 0.6 | 1 |
| Experience | Defection count | HRC | COM | vs | ROU | 1.09 | 0.76 | 0.61 | 0.96 |
| Experience | Defection count | HRC | COM | vs | ROU | 1.18 | 0.76 | 0.62 | 0.92 |
| Experience | Defection count | HRC | COM | vs | ROU | 1.27 | 0.75 | 0.62 | 0.9 |
| Experience | Defection count | HRC | COM | vs | ROU | 1.36 | 0.74 | 0.61 | 0.89 |
| Experience | Defection count | HRC | COM | vs | ROU | 1.45 | 0.73 | 0.6 | 0.89 |
| Experience | Defection count | HRC | COM | vs | ROU | 1.55 | 0.72 | 0.58 | 0.9 |
| Experience | Defection count | HRC | COM | vs | ROU | 1.64 | 0.71 | 0.56 | 0.92 |
| Experience | Defection count | HRC | COM | vs | ROU | 1.73 | 0.71 | 0.53 | 0.94 |
| Experience | Defection count | HRC | COM | vs | ROU | 1.82 | 0.7 | 0.51 | 0.97 |
| Experience | Defection count | HRC | COM | vs | ROU | 1.91 | 0.69 | 0.48 | 1 |

**Task Training**

**Instructions for the experimenter (not showed to the participant):**

The training tutorials of the tasks are on the training laptop. Make sure that the participants understand the instructions, ask if they understood everything or if they have any further questions. During the tutorials the investigator should point out the limited timing of the trials/decision situations. They also should point out that the participant will receive money depending on his performance during the PD (only a proportion). The investigator should explain that while no money is earned in the training session, money can be earned only in the actual experiment. Additionally, mention that the participant will play against another person who will do the task in another room at IBEB. After the PD tutorial, participants should do the test quiz below to see if they understood the instructions.

**Training quiz (to be filled by the participant):**
